# Pharmacological inhibition of hematopoietic progenitor kinase 1 positively regulates T-cell function

**DOI:** 10.1371/journal.pone.0243145

**Published:** 2020-12-03

**Authors:** Yun Wang, Kelvin Zhang, Peter Georgiev, Steven Wells, Haiyan Xu, Brian M. Lacey, Zangwei Xu, Jason Laskey, Robbie Mcleod, Joey L. Methot, Mark Bittinger, Alexander Pasternak, Sheila Ranganath

**Affiliations:** 1 Department of Oncology Early Discovery, Merck & Co., Inc., Boston, Massachusetts, United States of America; 2 Department of Genetics and Pharmacogenomics, Merck & Co., Inc., Boston, Massachusetts, United States of America; 3 Department of Quantitative Biosciences, Merck & Co., Inc., Boston, Massachusetts, United States of America; 4 Department of Discovery Chemistry, Merck & Co., Inc., Boston, Massachusetts, United States of America; Universite Paris-Sud, FRANCE

## Abstract

Hematopoietic progenitor kinase 1 (HPK1), a hematopoietic cell-specific Ste20-related serine/threonine kinase, is a negative regulator of signal transduction in immune cells, including T cells, B cells, and dendritic cells (DCs). In mice, HPK1 deficiency subverts inhibition of the anti-tumor immune response and is associated with functional augmentation of anti-tumor T cells. We have used a potent, small molecule HPK1 inhibitor, Compound 1, to investigate the effects of pharmacological intervention of HPK1 kinase activity in immune cells. Compound 1 enhanced Th1 cytokine production in T cells and fully reverted immune suppression imposed by the prostaglandin E_2_ (PGE_2_) and adenosine pathways in human T cells. Moreover, the combination of Compound 1 with pembrolizumab, a humanized monoclonal antibody against the programmed cell death protein 1 (PD-1), demonstrated a synergistic effect, resulting in enhanced interferon (IFN)-γ production. Collectively, our results suggest that blocking HPK1 kinase activity with small molecule inhibitors alone or in combination with checkpoint blockade may be an attractive approach for the immunotherapy of cancer.

## Introduction

The immune system plays an indispensable role in the maintenance of cellular homeostasis and active inhibition of tumorigenesis. However, as tumors arise, they develop various immunosuppressive mechanisms to bypass immune surveillance and evade the immune system [1]. Recently, many advances have been made in the field of immune-oncology (IO) therapy, the aim of which is to restore or augment an anti-tumor immune response [2]. For example, therapeutic antibodies targeting T-cell inhibitory checkpoint proteins such as cytotoxic T-lymphocyte-associated protein 4 (CTLA-4) and programmed cell death protein 1 (PD-1) have been demonstrated to be effective approaches to restore or enhance T-cell function and evoke a robust anti-tumor immune response [3]. To date, 6 monoclonal antibodies (mAbs) targeting PD-L1 (eg, Keytruda and Opdivo) and 1 mAb targeting cytotoxic T lymphocyte antigen 4 (CTLA-4) (Ipilimumab) have been approved by the US Food and Drug Administration to treat multiple types of cancers [4]. However, many patients show primary, adaptive, or acquired resistance to the treatment, potentially because of tumor heterogeneity or suppressive factors in the tumor microenvironment (TME), which dampen T-cell effector function [5]. In addition, blocking mAb therapies are restricted to negative regulators that are present on the surface of T cells, which limits their utility [6]. Therefore, development of small molecule drugs to target intracellular negative regulators of T cell function may represent a conceptually novel approach to bridge this gap.

Hematopoietic progenitor kinase 1 (HPK1), also named mitogen-activated protein kinase kinase kinase kinase 1 (MAP4K1), is a serine/threonine protein kinase with expression restricted to the hematopoietic compartment [7]. HPK1 is involved in modulating various downstream signaling pathways, including c-Jun N-terminal kinase (JNK), activator protein 1(AP-1), and nuclear factor-κB (NF-κB) [7–9]. These pathways regulate cellular proliferation, apoptosis, and immune cell activation, thereby contributing to the pathogenesis of autoimmune diseases, such as systemic lupus erythematosus (SLE), and cancers [6]. T-cell receptor (TCR) or B-cell receptor (BCR) signaling activates HPK1 by inducing HPK1/lymphocyte cytosolic protein 2 (SLP-76) or HPK1/B cell linker protein (BLNK) interaction in T cells and B cells, respectively [10, 11]. Activated HPK1 then phosphorylates SLP-76 at Ser376 in T cells or BLNK at Thr-152 in B cells to mediate 14-3-3 binding, thus negatively regulating TCR and BCR signaling [10–12]. HPK1-/- mouse T cells demonstrated increased proliferation and elevation of Th1 cytokines, interferon (IFN)-γ, and interleukin (IL)-2 in response to anti-CD3/CD28 [13–16]. Deletion of HPK-1 in mice has effects beyond antigen receptor signaling pathways; HPK1-/- bone marrow-derived DCs (BMDC) expressed higher levels of co-stimulatory molecules and pro-inflammatory cytokines and enhanced antigen presentation capacity [15–17], which is consistent with *ex vivo* evidence for anti-tumor activity in HPK1 null T cells and DCs. Studies with HPK1 kinase dead knock-in mice demonstrated decreased tumor growth and enhanced αPD-L1 efficacy [6, 16]. Further analysis of immune cell profiles from the TME confirmed that loss of HPK1 activity enhanced effector T-cell function and reduced numbers of immunosuppressive Foxp3+ regulatory T cells [6, 15]. In addition, HPK1 signal transduction impinges upon pathways activated by the immunosuppressive agonists prostaglandin E_2_ (PGE_2_) and adenosine [18, 19], reversing the effects of molecules that are key components of TME inhibitory mechanisms [20, 21]. Taken together, loss of HPK1 function promoted anti-tumor activity and demonstrated promising efficacy in pre-clinical models [6, 15, 16]. However, it remains unknown whether pharmacological inhibition of HPK1 by small molecules will recapitulate the benefits of genetic deletions.

In this study, we investigated the mechanism of Compound 1, a potent HPK1 inhibitor, in multiple immune cells and demonstrated that pharmacological inhibition of HPK1 enhanced T-cell functions. The HPK1 inhibitor demonstrated synergistic effects with pembrolizumab on IFN-γ production, suggesting its potential for anti-tumor immunotherapy alone or in combination with anti-PD1 blockade.

## Materials and methods

### Mice and reagents

Six- to eight-week-old female wild-type, OT-1 and OT-II mice on a C57BL/6 background were purchased from The Jackson Laboratory (Bar Harbor, ME). All animal procedures were approved by the Institutional Animal Care and Use Committee of Merck & Co., Inc., Kenilworth, NJ, USA.

The pharmacologic HPK1 inhibitor Compound 1 was synthesized according to the methods described in Genentech patent application WO 2018183964 A1. Pembrolizumab and IgG4 isotype were generated by Merck & Co., Inc., Kenilworth, NJ, USA. lipopolysaccharide (LPS) (Sigma-Aldrich, St.Louis, MO), forskolin (FSK) (Sigma-Aldrich, St.Louis, MO), PGE_2_ (Cayman Chemical, Ann Arbor, MI), ovalbumin (OVA) peptides (OVA_257-264_ and OVA_323-339_) (Sigma-Aldrich, St.Louis, MO), and 5’-N-ethylcarboxamido adenosine (NECA) (Tocris, Bristol, UK) were prepared and used according to the manufacturer’s instructions. Anti-human CD3 (hCD3) (Clone OKT3), anti-human CD28 (hCD28) (Clone CD28.2), anti-mouse CD3 (mCD3) (Clone 145-2C11), anti-mouse CD28 (mCD28) (Clone 37.51), and carboxyfluorescein succinimidyl ester (CFSE) cell labeling kit were from Thermo Fisher Scientific (Waltham, MA). Mouse granulocyte macrophage-colony stimulating factor (GM-CSF) and mouse IL-4 were obtained from Peprotech (NJ, USA). All flow antibodies were purchased from Biolegend or BD Bioscience (San Diego, CA).

### Single cell RNA-sequencing data analysis

The single cell RNA-seq data set of human peripheral blood mononuclear cells (hPBMCs) from a healthy donor was downloaded from the 10x Genomics website: https://support.10xgenomics.com/single-cell-gene-expression/datasets/2.1.0/pbmc8k.

The dataset consists of 8381 cells sequenced on Illumina Hiseq4000, with approximately 92,000 reads per cell. Data analysis and visualization were performed using Seurat [22]. Briefly, cells were first filtered with the unique feature counts/cell greater than 200 and less than 2,500 and less than 5% mitochondrial counts/cell. The filtered data were then normalized using a global-scaling normalization method (LogNormalize). The data were further processed with the principal component analysis (PCA) using the top 2,000 most variable genes. Finally, a graph-based clustering approach was applied to the previously defined dimensionality of the data (the first 10 principal components). For the visualization, the t-distributed stochastic neighbor embedding (tSNE) method was employed on the top 10 principal components. The following canonical cell type markers were used to define each cell population: IL7R/CCR7/CD3E/CD4 for naïve CD4+T cells, IL7R/S100A4/CD3E/CD4 for memory CD4+T cells, CD14/LYZ for CD14+ monocytes, MS4A1 for B cells, CD8α/CD3E/CD4 for CD8+ T cells, FCGR3A/MS4A7 for FCGR3A+ monocytes, GNLY/NKG7 for natural killer (NK) cells and FCER1A/CST3 for dendritic cells (DCs).CD8 clusters were not big enough for further classification into subsets.

### T-cell activation and proliferation

Cryopreserved human PBMCs were either directly purchased from HemaCare (CAT# PB009C-1, Northridge, CA) or were prepared by density centrifugation with Ficoll from Leukopaks (HemaCare, CAT# PB001LCLP, Northridge, CA). According to the vendor, leukopaks were collected in HemaCare’s FDA-registered collection centers following cGMP and cGTP collection guidelines from Institutional Review Boards (IRB)-consented healthy human donors. All the human sample related procedures were approved by Western Institutional Review Board (WIRB)–compliant Merck IRB at Merck & Co., Inc., Kenilworth, NJ, USA. Naïve CD4 (CD4+CD45RA+CCR7+), memory CD4 (CD4+CD45RO+ CD45RA−CCR7−), naïve CD8 (CD8+CD45RA+CCR7+) and memory CD8 (CD8+CD45RO+ CD45RA−CCR7−) were sorted using a BD FACS Aria II instrument according to a previous study [23] or directly purchased from HemaCare (Northridge, CA) whereas bulk human CD4+(hCD4+) and human CD8+ (hCD8+) T cells were isolated from hPBMCs using CD4+ and CD8+ T-cell isolation kits (Miltenyi Biotech, Bergisch Gladbach, Germany) following the manufacturer’s instructions. Purified lymphocytes were treated with various concentrations of HPK1 inhibitor Compound 1 alone or with PGE_2_, NECA, or FSK, and then stimulated with plate-bound anti-hCD3/hCD28 for 24 hours in complete medium (cRPMI). For proliferation studies, T cells were loaded with CFSE using the cell trace CFSE proliferation kit (Thermo Fisher Scientific, Waltham, MA) and stimulated with plate-bound anti-hCD3/anti-hCD28 mAbs at 0.25μg/mL in cRPMI for 72 hours, followed by blockade of CD16/CD32 antibody and cell surface staining. For HPK1 inhibitor and pembrolizumab combination experiments, hPBMCs were treated with Compound 1 with and without pembrolizumab and stimulated with plate-bound anti-hCD3/anti-hCD28 for 24 hours, 48 hours, and 72 hours. For mouse T-cell assay, mouse splenocytes were extracted from fresh spleens and passed through a 70μm cell strainer. After erythrocyte removal with ACK lysis buffer (Thermo Fisher Scientific, Waltham, MA), splenocytes from OT-1 and OT-II mice were treated with Compound 1 for 1 hour before being stimulated with OVA_257-264_ or OVA_323-339_ peptide in complete medium for 24 hours and 72 hours. Media were collected for cytokine analysis, and cells were analyzed by flow cytometry. Mouse T-cell proliferation was determined by intracellular Ki-67 staining after stimulation.

### Dendritic cell activation and analysis

Mouse bone marrow cells were harvested from the femurs of C57/BL6 mice and cultured in cRPMI with recombinant 20 ng/mL mouse GM-CSF and 20 ng/mL mouse IL-4 for 6 days to generate immature DCs. Human DCs purchased from HemaCare and immature mouse DCs generated in house were pretreated with Compound 1 for 1 hour and then stimulated with LPS. After 18 to 24 hours of incubation, media were collected for cytokine measurement, and cells were stained with fluorescence-labeled antibodies against CD11c, mI-A/I-E, mCD40, mCD86, hCD80, hCD83 and hCD86 for flow cytometry analysis.

### Multiplex cytokine analysis

Secreted cytokines from T lymphocytes and DCs in the supernatant were measured by a V-plex pro-inflammatory panel 1 mouse or human kit from Meso Scale Discovery (MSD) (Gaithersburg, MD). The mouse kit measures IFN-γ, IL-1β, IL-2, IL-4, IL-5, IL-6, IL-10, IL-12p70, KC/GRO, TNF-α and the human kit measures IFN-γ, IL-1β, IL-2, IL-4, IL-6, IL-8, IL-10, IL-12p70, IL-13, and TNF-α. MSD plates were analyzed on the MS2400 imager (MSD, Gaithersburg, MD). All assays and analysis were performed according to the manufacturer’s instructions.

### Flow cytometry

The following antibodies were used: anti-human CCR7 (hCCR7)(Clone 4B12), anti-human CD45RA (hCD45RA)(Clone HI100), anti-human CD45RO (hCD45RO)(Clone UCHL1), anti-human CD4 (hCD4) (Clone RPTA-4), anti-human CD8 (hCD8) (Clone RPTA-8), anti-human CD25 (hCD25) (Clone M-A251), anti-human CD69 (hCD69) (Clone FN50), anti-human CD71 (hCD71) (Clone CYIG4), anti-human CD80 (hCD80)(Clone 2D10), anti-human CD83(hCD83)(Clone HB15e), anti-human CD86(hCD86)(Clone BU63), anti-mouse CD4 (mCD4) (Clone GK1.5), anti-mouse CD8 (mCD8) (Clone 53–6.7), anti-mouse CD86 (mCD86) (Clone GL-1), anti-mouse I-A/I-E (mI-A/I-E) (Clone M5/114.15), anti-mouse CD40 (mCD40)(Clone HM40-3) and anti-mouse Ki-67 (Clone 16A8). Cells were incubated with fixable viability stain 510 (FVS510) or 780 (FVS780) (BD Biosciences, San Diego, CA), followed by blocking with anti-mouse CD16/CD32 antibody (Clone 2.4G2) or human BD Fc Block (BD Biosciences, San Diego, CA) for 10 minutes. Thereafter, cells were stained with fluorochrome-conjugated surface antibodies by incubation for 30 minutes in cell-staining buffer (Biolegend, San Diego, CA). eBioscience™ Foxp3 / Transcription Factor Staining Buffer Set was used for intracellular staining (Thermo Fisher Scientific, Waltham, MA). Fixation & Permeabilization and staining were performed according to manufacturer’s instructions. Stained samples were acquired on a Fortessa cytometer with DIVA software (BD Biosciences, San Diego, CA), and the raw data were analyzed using FCS Express (De Novo Software, Pasadena, CA).

### Statistical analysis

Data were plotted as mean ± standard deviation (SD). Paired two-tailed Student’s t-test or one-way ANOVA with Dunnett test (compared to vehicle control or Compound 1 0μM group) was performed using GraphPad Prism (GraphPad Software, La Jolla, CA). Differences were considered at P<0.05. *P<0.05, **P<0.01, ***P<0.001, and ****P<0.0001.

## Results

### HPK1 and its substrates are widely expressed in subsets of immune cells

**Fig 1:**
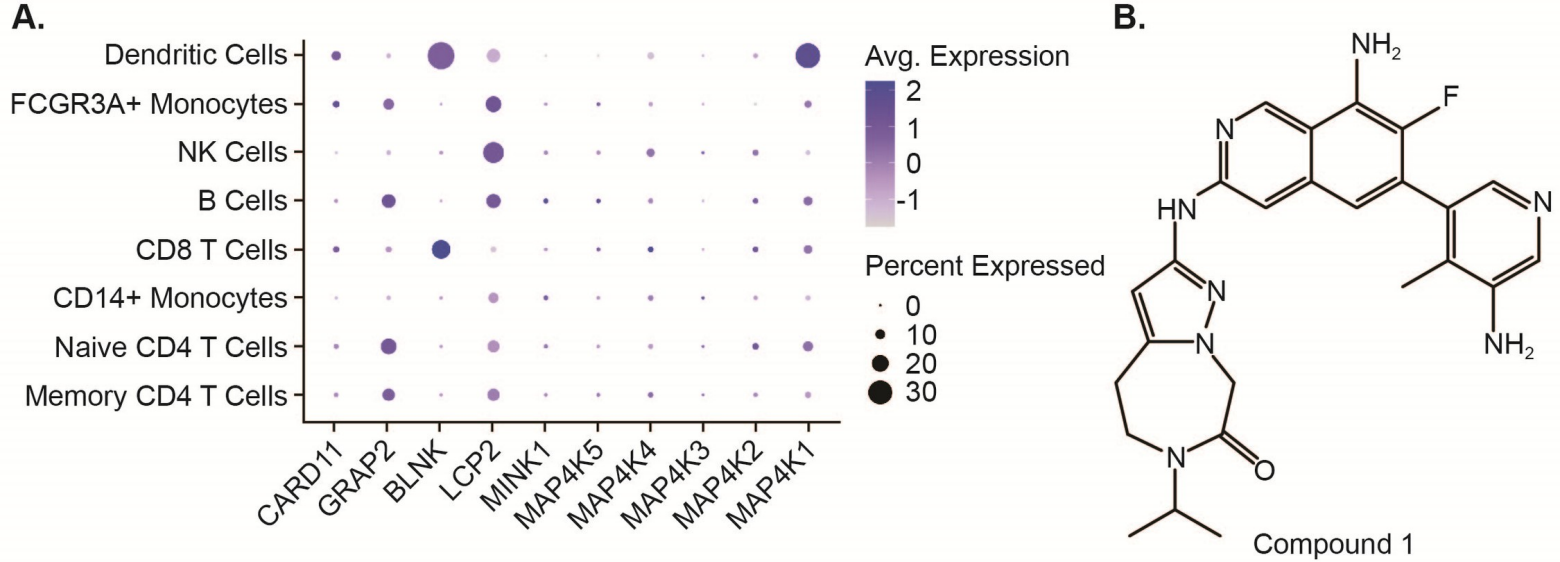
Expression of HPK1 in immune cells. A. Dot plots demonstrating expression patterns of MAP4K family members and substrates for each immune cell type in hPBMCs. Each dot represents an individual cell type, and dot size denotes percentage of cells expressing the marker. B. Structure of Compound 1 used in the study.

MAP4Ks, members of the Ste20-like family of serine/threonine kinases, include MAP4K1 (HPK1), MAP4K2, MAP4K3, MAP4K4, MAP4K5, and MAP4K6 (MINK1) [24]. HPK1 is reported to interact with various substrates and adaptor proteins, such as lymphocyte cytosolic protein 2 (LCP2, also called SLP-76), BLNK, GRB-related adaptor protein 2 (GRAP2), and caspase recruitment domain family member 11 (CARD11) [24]. These adaptor proteins play a crucial role in regulating the activation and function of HPK1 [24]. To understand the expression patterns of the MAPK4 family kinases and their substrates in immune cells, we conducted single cell RNA-sequencing (RNA-seq) analysis of a publicly available hPBMC data set. Relative to other MAPK4s, HPK1 was expressed in DCs most abundantly (Fig 1A). HPK1 was also expressed in other immune cells, such as B cells, NK cells, naïve CD4+ T cells, memory CD4+ T cells, CD8+ T cells, and monocytes (Fig 1A). Although the CD8+ T cell clusters were not sufficiently large for additional subset analysis, HPK1 expression in naïve CD4+ T cells, memory CD4+ T was very similar. We found that the mRNA expression pattern of MAP4K2 and MAP4K4 was similar to that of HPK1, while MAP4K3/5/6 was expressed at a much lower level in all analyzed cell types except DCs. A similar expression trend was observed for HPK1 substrates LCP2 (SLP-76), GRAP2, CARD11, and BLNK (Fig 1A). To evaluate the effect of pharmacological inhibition of HPK1, we used Compound 1 (Fig 1B), a selective HPK1 inhibitor discovered by Genentech [25].

Compound 1 was characterized by a biochemical assay and determined to have an IC50 of 0.0465 nM in inhibiting HPK1 activity as well as decreasing SLP-76 phosphorylation in a human pSLP-76 enzyme-linked immunosorbent assay (ELISA) at an IC_50_ lower than 0.02 μM [26]. These findings indicated that Compound 1 was potent in decreasing HPK1 activity [26]. Based on a biochemical screening assay against a panel of 265 kinases, Compound 1 had greater than 100-fold selectivity against 260 kinases, with the main off targets having less than 100-fold selectivity against leucine-rich repeat kinase 2 (LRRK2), MAP4K2, MAP4K3 and MAP4K5 [26].

### Pharmacological inhibition of HPK1 augments T cell activation and cytokine production

**Fig 2:**
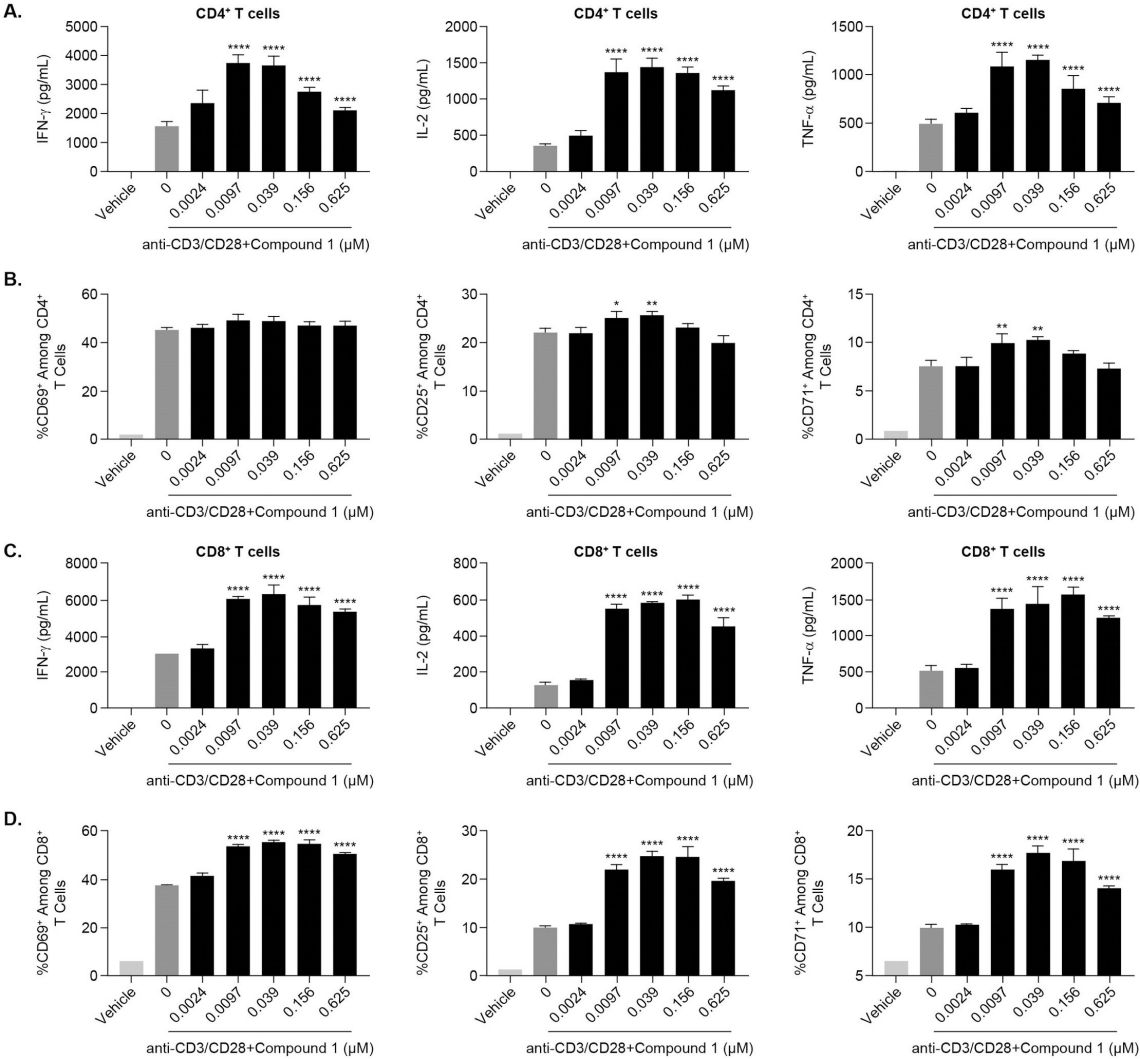
Effects of Compound 1 on anti-CD3/CD28-stimulated human CD4+ and CD8+ T cells. Human CD4+ T cells (A and B) and CD8+ T cells (C and D) were isolated from hPBMCs and stimulated with 0.25μg/mL anti-CD3 and 0.25μg/mL anti-CD28 for 24 hours in the presence or absence of Compound 1. IFN-γ, IL-2, and TNF-α secretion (A and C) was measured from supernatants using the Mesoscale Discovery (MSD) ELISA-based assay platform, and the frequency of CD25, CD69, and CD71 positive cells (B and D) among the hCD4 or hCD8 T cell compartments was assessed by flow cytometry. Data are shown from 1 experimental representative (triplicate treatment) of at least 3 independent experiments (different donors). *P<0.05, **P<0.01 ***P<0.001, ****P<0.0001, one-way ANOVA with Dunnett test analysis compared to anti-CD3/CD28 group.

Germline deletion of HPK1 enhances T-cell activation by increasing cytokine secretion and T-cell proliferation [13, 14]. To evaluate whether Compound 1 enhances T-cell function by disabling an internal checkpoint, CD4+ and CD8+ T cells were purified from healthy donor (hPBMCs) and stimulated *in-vitro* with anti-CD3/CD28 mAbs in the presence or absence of Compound 1 for 24 hours or 72 hours. CD4+ and CD8+ T cell viability was not affected by Compound 1 treatment at doses lower than 2.5μM in our study (S1A and S1B Fig). Activated CD4+ and CD8+ T cells treated with Compound 1 for 24 hours produced higher levels of IFN-γ, IL-2, and TNF-α as compared to untreated controls (Fig 2A and 2C) with a similar trend observed at 72 hours (S2A and S2B Fig). These results are consistent with the observation that T cells from HPK1 null mice express more IFN-γ and IL-2 as compared to wild-type control T cells [14, 16]. We also evaluated the expression of canonical T cell surface activation markers, including CD25, CD69, and CD71. We found that the expression of these activation markers was increased upon TCR stimulation and that this effect was augmented following treatment with Compound 1 (Fig 2B and 2D). CD8+ T cells exhibited a stronger response to Compound 1 as compared to CD4+ T cells, increasing at least 30% of activation marker expression as compared to TCR stimulation alone, beginning at the 0.01 μM concentration (Fig 2D and S3B Fig). However, in CD4+ T cells (Fig 2B and S3A Fig), treatment with 0.01 μM and 0.04 μM concentrations of Compound 1 led to a modest increase in the frequencies of CD25 and CD71 positive cells as compared to anti-CD3/CD28 alone, suggesting that Compound 1 may be differentially potent with respect to CD8+ vs CD4+ T cells. In addition, Compound 1 exerted a modest stimulatory effect on the proliferation of CD8+ T cells (S4B Fig, left panel) whereas no effect was observed in CD4+ T cells (S4A Fig, left panel).

**Fig 3:**
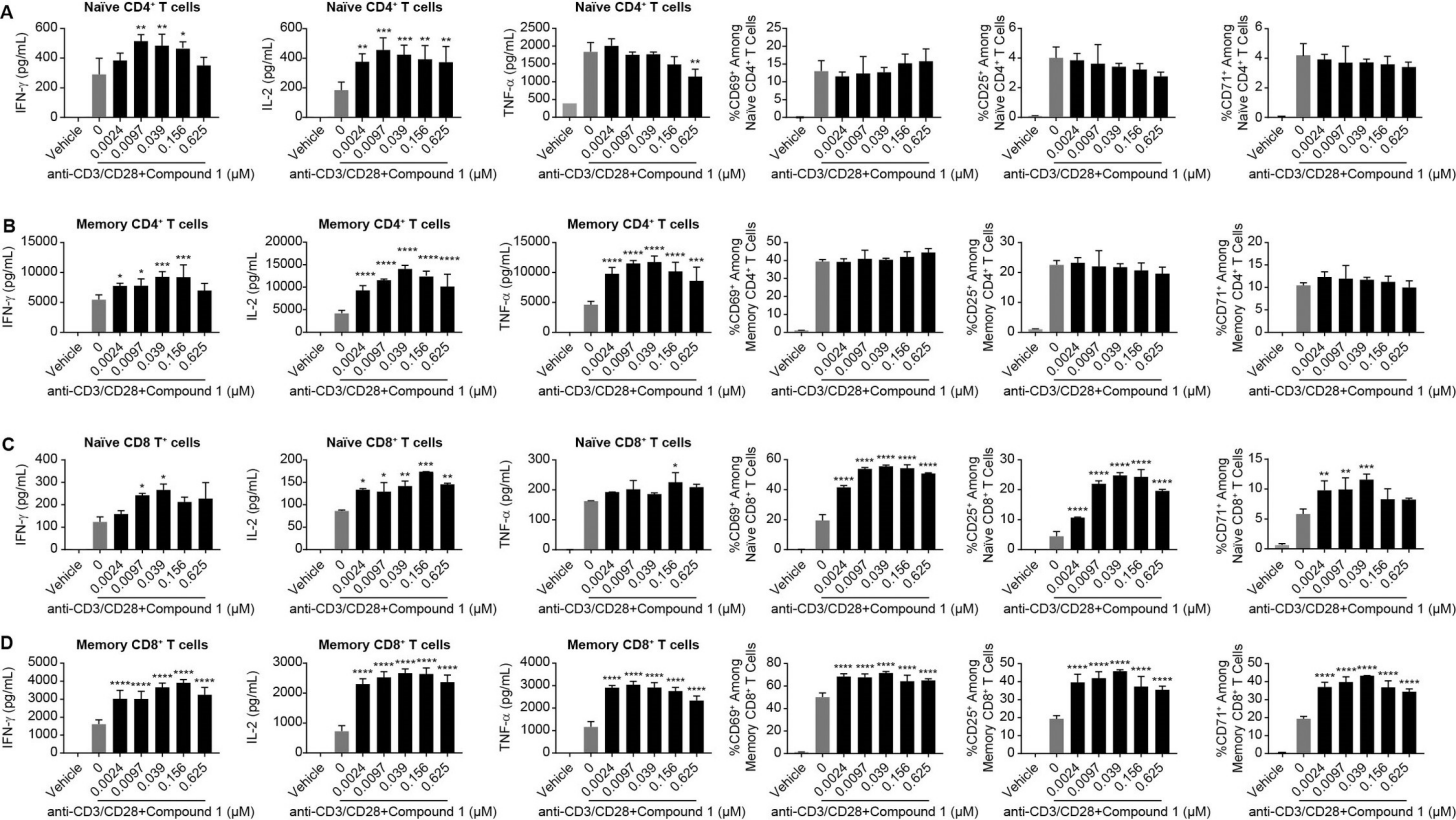
Effects of Compound 1 on anti-CD3/CD28-stimulated human naïve and memory T cells. Human naïve CD4+ T cells (A), memory CD4+ T cells (B), naïve CD8+ T cells (C) and memory CD8+ T cells (D) were sorted from hPBMC and stimulated with 0.25μg/ml anti-CD3 and anti-CD28 for 24h. IFN-γ, IL-2, and TNF-α secretion were measured from supernatants using the Mesoscale Discovery (MSD) ELISA-based assay platform and % of activation marker CD25, CD69 and CD71 among naïve or memory CD4+ or CD8+ T cells were determined by flow cytometry. Data are shown from 1 experimental representative (triplicate treatment) of at least 3 independent experiments (different donors). *P<0.05, **P<0.01 ***P<0.001, ****P<0.0001, one-way ANOVA with post-test analysis compared to anti-CD3/anti-CD28 group.

To determine whether Compound 1 exhibits differential effects within the CD4+ and CD8+ T cell compartments, sorted naïve CD4+, memory CD4+, naïve CD8+ and memory CD8+ T cells were stimulated in the presence or absence of Compound 1. In line with its stimulatory effects in bulk CD4+ and CD8+ T lymphocytes (Fig 2A–2D and S3A and S3B Fig), Compound 1 significantly increased IFN-γ and IL-2 production in both naïve and memory CD4+/CD8+ T cells (Fig 3A–3D) whereas its stimulatory effects on TNF-α production were restricted to the memory CD4+/CD8+ T cell subset (Fig 3A–3D). The frequency of CD25, CD69, and CD71 positive cells was significantly enhanced by Compound 1 treatment in naïve and memory CD8+ T cells but not in naïve and memory CD4+ T cells (Fig 3A–3D), consistent with observations in bulk CD4+/CD8+ T cells (Fig 2). Moreover, the effects of Compound 1 on bulk CD8+ T cell proliferative responses (S4B Fig, left panel) were recapitulated using naïve and memory CD8+ T cells (S4B Fig, middle and right panel). Compound 1 did not exhibit any effect on naïve and memory CD4+ T cell proliferation (S4A Fig, middle and right panel), which is also supported by observations in bulk CD4+ T cells (S4A Fig, left panel). Taken together, these results extend on our initial analysis and further validate the activity of Compound 1 on human T cell functional responses.

**Fig 4:**
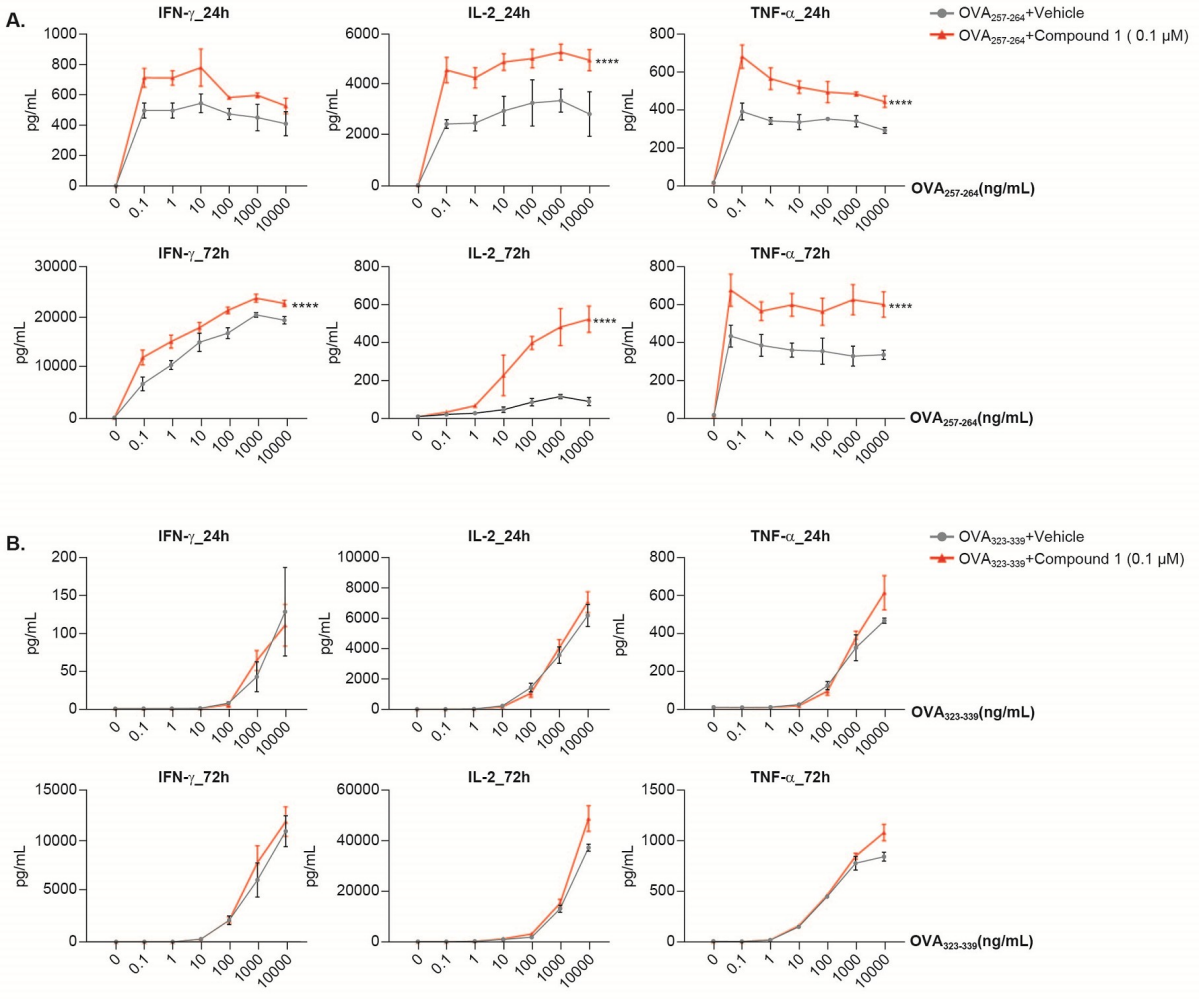
Compound 1 effect on OVA peptide-stimulated mouse splenocytes. Splenocytes from OT-1 mice (A) or OT-II (B) mice were treated with Compound 1 at 0.1 μM and stimulated with various concentrations of OVA_257-264_ (A) or OVA_323-339_ (B) for 24 and 72 hours. IFN-γ, IL-2, and TNF-α level in supernatant was measured using the Mesoscale Discovery (MSD) ELISA-based assay platform. Data are shown from 1 experimental representative (quadruplicate treatment) of at least 3 independent experiments. ****P<0.0001, one-way ANOVA with Dunnett test analysis compared to OVA_257-264_ +vehicle or OVA_323-339_ +vehicle.

To further elucidate the effects of Compound 1 in CD4+ and CD8+ T cells, we evaluated the patterns of cytokine secretion and proliferation in murine T cells using mice harboring TCRs specific for ovalbumin (OVA). Flow cytometry analysis confirmed that CD8+ T cells were the predominant population in splenocytes from OT-1 mice and that only CD8+ T cells were specifically expanded by OVA_257-264_ following 72 hours of stimulation (S5A Fig). By contrast, CD4+ T cells were the main population in the spleen of OT-II mice, and OVA_323-339_ specifically induced CD4+ T-cell proliferation (S5B Fig). Splenocytes from OT-1 or OT-II mice were stimulated with various concentrations of OVA_257-264_ and OVA_323-339_ in the presence and absence of 0.1 μM Compound 1 for 24 and 72 hours. Compound 1 significantly increased IL-2 and TNF-α secretion as compared to OVA_257-264_ stimulation alone at both the 24- and 72-hour time point in CD8+ T cells (Fig 4A). By contrast, Compound 1 did not significantly affect OVA_323-339_-induced IFN-γ, IL-2, and TNF-α secretion in CD4+ T cells at 24 hours and 72 hours (Fig 4B), similarly confirming a more potent effect of Compound 1 in CD8+ T cells relative to CD4+ T cells. The frequency of Ki-67 positive CD4+ T cells did not change after treatment with Compound 1 whereas there was a modest increase in frequency of Ki-67 positive CD8+ T cells (S4C Fig), which is consistent with the effects of Compound 1 on TCR-induced human T-cell proliferation (S4A and S4B Fig).

### Synergistic effects of the HPK1 inhibitor Compound 1 and pembrolizumab on IFN-γ release in human PBMCs

**Fig 5:**
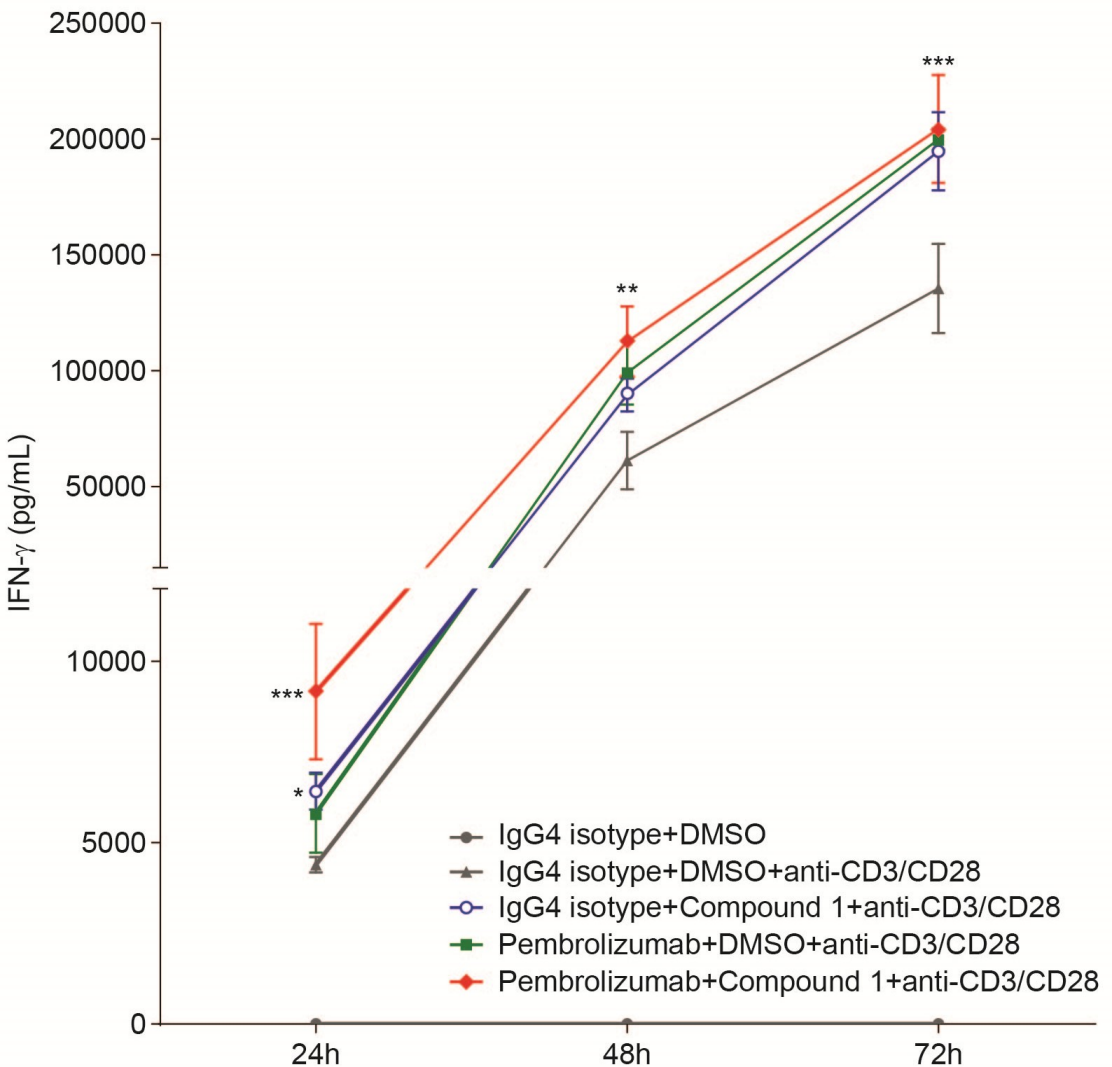
Synergistic effect of Compound 1 and pembrolizumab on IFN-γ production from human PBMCs. Human PBMCs were treated with 0.04 μM Compound 1 with or without pembrolizumab and stimulated with plate bound anti-CD3/CD28 at a concentration of 0.25 μg/mL for 24, 48, and 72 hours. IFN-γ secretion in supernatants was measured using the Mesoscale Discovery (MSD) ELISA-based assay platform. Data are shown from 1 experimental representative (quadruplicate treatment) of at least 3 independent experiments. *P<0.05, **P<0.01 ***P<0.001, ****P<0.0001, one-way ANOVA with Dunnett test analysis compared to anti-CD3/CD28 group.

A recent study demonstrated that mice containing a germline mutation in HPK1 that abolishes its kinase activity and harboring an implanted tumor, exhibited an enhanced response to anti-PD-L1 immunotherapy as compared to tumor bearing wild type mice, suggesting that inhibition of HPK1 in T lymphocytes may potentiate the activity of checkpoint inhibitors [16, 27]. To examine whether HPK1 blockade by Compound 1 could synergize with pembrolizumab *in vitro*, hPBMCs were stimulated with anti-CD3/CD28 mAbs in the presence or absence of Compound 1 and pembrolizumab, and IFN-γ concentrations were assessed in the media following 24, 48 and 72 hours of treatment. Compound 1 alone significantly increased IFN-γ secretion, while pembrolizumab did not affect IFN-γ levels at the 24-hour time point (Fig 5). The combination of Compound 1 and pembrolizumab led to increased IFN-γ production at the 24-hour timepoint. Although treatment with either Compound 1 or pembrolizumab significantly enhanced IFN-γ production at the 48- and 72-hour time points, the combination of Compound 1 and pembrolizumab did not increase IFN-γ levels. These results indicated that pharmacological inhibition of HPK1 by Compound 1 led to transient T-cell activation but was not sufficient to elicit a durable combination effect.

### HPK1 inhibitor Compound 1 reverses cAMP-induced suppression of T cells

**Fig 6:**
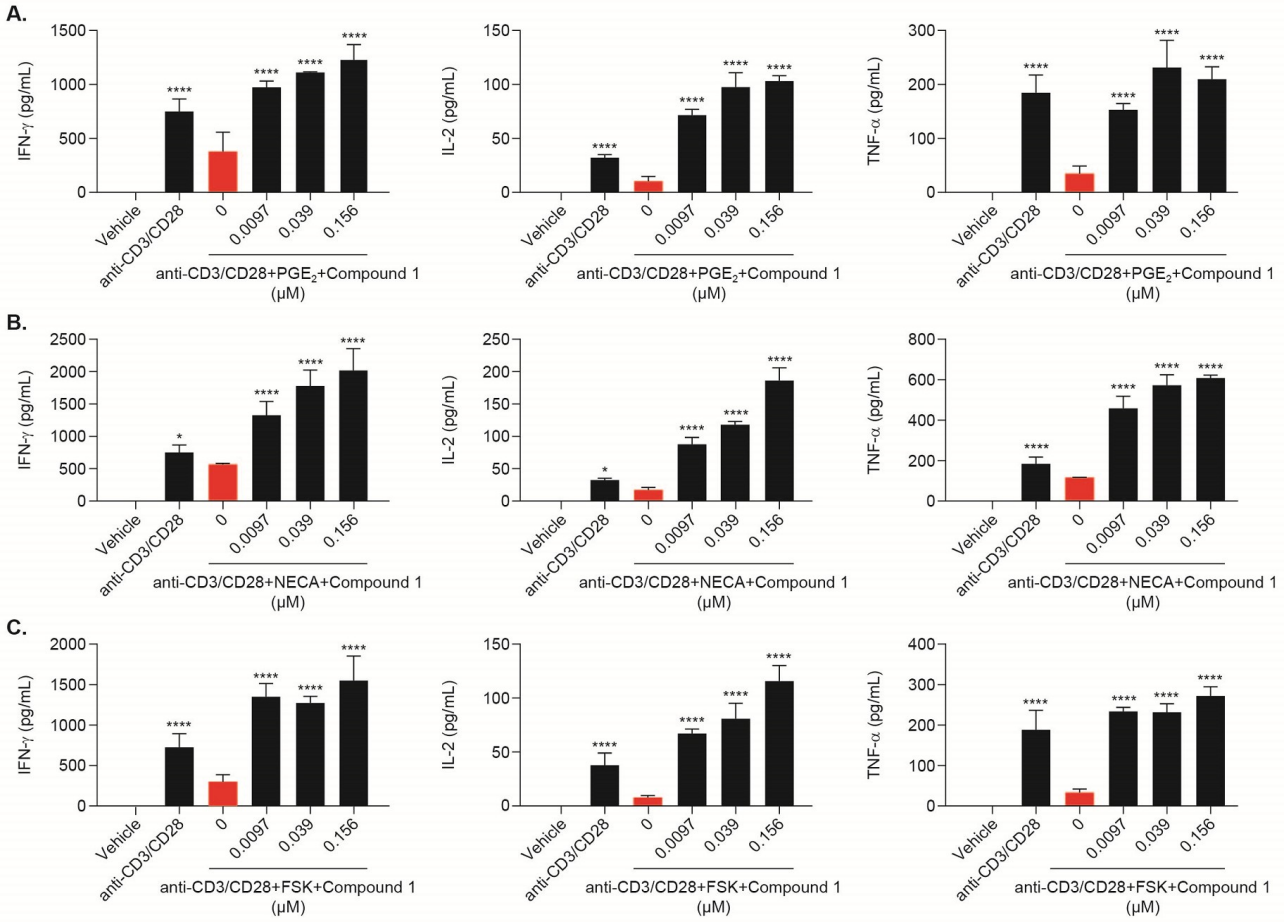
Compound 1 restored cAMP-suppressed cytokines in anti-CD3/CD28-stimulated human CD8+ T cells. Human CD8+ T cells were isolated from hPBMCs and treated with Compound 1 with and without PGE_2_ (A), NECA (B), or FSK (C), and then stimulated with 0.5μg/mL anti-CD3 and anti-CD28 for 24 hours. IFN-γ, IL-2, and TNF-α secretion in the supernatant was measured using the Mesoscale Discovery (MSD) ELISA-based assay platform. Data are shown from 1 experimental representative (triplicate treatment) of at least 3 independent experiments (3 different donors). *P<0.05, **P<0.01 ***P<0.001, ****P<0.0001, one-way ANOVA with Dunnett test analysis compared to anti-CD3/CD28+/PGE_2_/NECA/FSK+0 μM Compound 1 group.

In addition to TCR-induced activation of HPK1, the intracellular cAMP-dependent protein kinase A (PKA) pathway can alternatively induce robust HPK1 kinase activity [19]. In the TME, suppressive factors such as PGE_2_ and adenosine activate the intracellular cAMP-PKA pathway through binding to G protein-coupled receptors (GPCRs). Increased PKA signaling, in turn, activates HPK1 and leads to tumor resistance [18, 19]. HPK1-deficient T cells are resistant to immune suppression exerted by PGE_2_ and adenosine [14, 20]. In this study, we treated hCD8+ T cells with various concentrations of Compound 1 and stimulated these cells with anti-CD3/CD28 mAb in the presence of PGE_2_ or NECA, a stable adenosine analogue. PGE_2_ (Fig 6A) and NECA (Fig 6B) dramatically decreased IFN-γ, IL-2, and TNF-α secretion 24 hours following TCR stimulation. Concurrent pharmacological inhibition of HPK1 by Compound 1 fully reversed PGE_2_ and NECA mediated suppression and additionally stimulated increased cytokine concentrations as compared to anti-CD3/CD28 mAb alone (Fig 6A and 6B). In addition to PGE_2_ and adenosine, which induced cAMP-PKA dependent signaling through GPCR-agonist binding, the adenylate cyclase activator forskolin (FSK) has been shown to directly increase intracellular cAMP and inhibit T-cell proliferation [28]. We found that Compound 1 similarly reversed FSK-mediated suppression in CD8+ T cells (Fig 6C), suggesting that inhibition of HPK1 could restore cAMP-mediated immune suppression in T cells. Furthermore, Compound 1 was also able to restore PGE2/NECA/FSK-suppressed cytokine release in hCD4+ T cells (S6 Fig).

### HPK1 inhibitor Compound 1 does not enhance dendritic cell function

**Fig 7:**
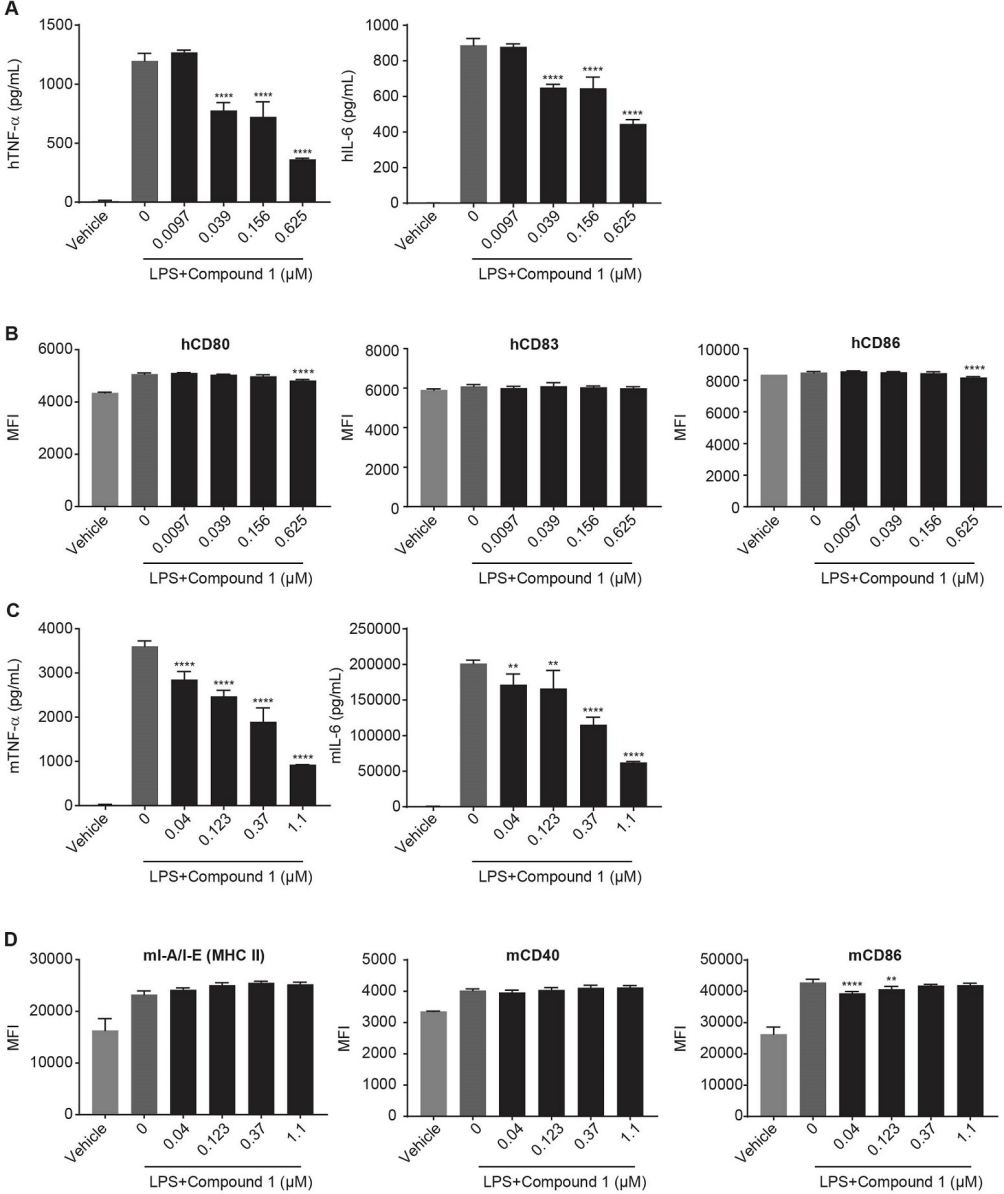
Compound 1 effect on cytokine release and activation marker expression in dendritic cells. Human DCs (A and B) or bone marrow-derived mouse DCs (C and D) were pretreated with Compound 1 for 1 hour, followed by stimulation with 0.2 μg/mL LPS for 24 hours. TNF-α and IL-6 secretion in the media was analyzed using the Mesoscale Discovery (MSD) ELISA-based assay platform (A and C). Expression of surface markers was analyzed by flow cytometry. Geometric mean fluorescent intensity (MFI) of hCD83, hCD80, hCD86 in hDC and MFI of mI-A/I-E (MHC II), mCD40 and mCD86 in mDC was shown in B and D. The data shown are representative (triplicate treatment) of 3 independent experiments. *P<0.05, **P<0.01 ***P<0.001, ****P<0.0001, one-way ANOVA with Dunnett test analysis compared to LPS group.

HPK1 is highly expressed in DCs (Fig 1A), and HPK1 deficiency in DCs results in increased pro-inflammatory cytokine secretion and heightened expression of co-stimulatory molecules [16, 17]. To investigate the role of pharmacological HPK1 inhibition by Compound 1 in DCs, we treated mouse bone marrow-derived DCs or human monocyte-derived DCs with Compound 1 and stimulated them with LPS for 24 hours. Supernatants were collected for cytokine measurements and cells were stained with anti-mCD40, anti-mCD86, and anti-mI-A/I-E or anti-hCD80, anti-hCD83 and anti-hCD86. After 18 to 24 hours of incubation, both human and mouse DCs produced high levels of TNF-α and IL-6 (Fig 7A and 7C) and had a marked increase in surface expression of hCD80, hCD86, hCD83 or mI-A/I-E (MHC II), mCD40 and mCD86 respectively as compared to vehicle-treated controls (Fig 7B and 7D). However, neither cytokine secretion (Fig 7A and 7C) nor activation marker expression was enhanced by pretreatment with Compound 1 (Fig 7B and 7D). Instead, Compound 1 decreased TNF-α and IL-6 (Fig 7A and 7C) secretion while co-stimulatory molecule expression was not affected after Compound 1 treatment (Fig 7B and 7D). We also examined the role of Compound 1 in the development of DCs during *in-vitro* mouse DC differentiation, but we did not observe any effects in Compound 1 treated samples as compared to untreated controls (S7 Fig).

## Discussion

The approval of immune checkpoint inhibitors has successfully expanded the options to treat many types of cancer during the past 10 years [4]. However, only a small number of patients benefit from these therapeutics, as cancer heterogeneity and the evolving suppressive TME render many cancers resistant to immunotherapy. This lack of response often correlates with poor immune cell infiltration of the tumor, although some well infiltrated tumors still fail to respond to immunotherapy [29]. This finding has spurred an urgent need to investigate new combinations that enhance the activity of T cells [5]. Combination therapy has emerged as the next strategy to increase response rates, and many classes of compounds are being combined with anti-PD-1 with the hopes of improving response rates. Anti-PD-1 combinations with standard of care therapies, including chemotherapy and vascular endothelial growth factor (VEGF) inhibitors have improved responses to anti-PD-1 blockade in numerous cancer types [30]. Another class of combination therapy, IO/IO combinations represent a conceptually novel shift in cancer immunotherapy treatment paradigms. For instance, the first IO/IO combination of Ipilimumab and Nivolumab (Ipi/Nivo) in melanoma and NSCLC, two well infiltrated tumor types, results in improved anti-tumor activity as compared to each respective monotherapy [31] although with increased side effects [32]. Bempegaldesleukin, a PEGylated IL-2, combined with nivolumab is showing promise in several clinical trials [33, 34]. In the journey to discover additional IO/IO combinations, the nonclassical MAPK family member HPK1, stands out in virtue of its capacity to regulate TCR-induced NK-κB activation and JNK signaling pathways, which are required for induction of an adaptive immune response [35, 36]. Blockade of HPK1 signaling has been suggested as a potential adjuvant to immune checkpoint blockade [35–37]. Moreover, loss of HPK1 kinase function in preclinical models of cancer results in enhanced anti-tumor T cell activity, suggesting HPK1 attenuates T cell functional responses [6, 15, 16]. However, the translational relevance of pharmacological inhibition of HPK1 remains to be fully elucidated. By using a small molecule inhibitor, Compound 1, to disrupt HPK1 activity, we evaluated its mechanism of action in various immune cell populations.

Consistent with previous studies with HPK1 null or HPK1 kinase-deficient cells [11, 14], Compound 1 augmented T-cell activation *in vitro*. In our study, we demonstrated that HPK1 inhibition by Compound 1 augmented TCR-induced cytokine and activation marker expression in human T cells. Interestingly, we observed a stronger stimulatory effect on activation marker expression in hCD8+ T cells as compared to hCD4+ T cells, although the HPK1/adaptor protein expression profiles in CD4+ and CD8+ T cells were similar. The preferential effect of Compound 1 in augmenting CD8+ vs CD4+ T-cell activation was also observed in mouse T cells upon antigen-specific stimulation. Moreover, Compound 1 also mediated substantially better rescue of CD8+ T cells in the context of immunosuppressive agents that subvert anti-tumor T cell responses, including extracellular adenosine and PGE_2_. Following TCR activation in the presence of Compound 1, CD8+ T cells exhibited a significant albeit modest increase in proliferation as assessed by CFSE dilution. These results are inconsistent with several previous studies demonstrating that HPK1-deficient T cells exhibit markedly enhanced proliferation as compared to HPK-1 sufficient control T cells [6, 14–16]. These observations suggest that pharmacological inhibition of HPK1 by Compound 1 does not fully recapitulate the level of proliferation augmentation resulting from T-cell HPK1 knockout. One possible explanation for this discrepancy is that HPK1 plays a requisite role in restraining aberrant T cell activity during the differentiation and development of T lymphocytes which cannot be unleashed by transient pharmacological blockade of HPK. We do not discount the possibility that Compound 1 may not fully block HPK1 activity, whereby residual HPK1 activity in T cells is sufficient for inhibition of proliferation following TCR engagement but insufficient for suppression of cytokine secretion. Moreover, these inconsistencies might arise from off-target effects of Compound 1, which is characteristic of small molecule kinase inhibitors [38]. For instance, based on the biochemical selectivity assays, Compound 1 had off target effects on LRRK2, MAP4K2, MAP4K3, and MAP4K5, which might differentially influence its activity on T cells [26].

Loss of HPK1 kinase function has been reported to enhance anti-PD-L1 efficacy *in vivo* [16], but combination treatment with pembrolizumab and Compound 1 showed a modest synergistic effect on IFN-γ production during acute activation. The IFN-γ level at 72 hours was similar with both Compound 1 and pembrolizumab treatment, suggesting lack of a durable synergistic effect from each agent. However, given that a single treatment of pembrolizumab and Compound 1 results in similar IFN-γ concentrations at 72 hours, pharmacological inhibition of HPK1 may represent an alternative monotherapy approach for PD-1 resistant patients. For example, Hernandez S et al demonstrated that loss of HPK1 function promotes anti-tumor activity in a mouse model, which could be enhanced by concurrent administration of an anti-PD-L1 antibody, suggesting that these two inhibitory pathways are non-overlapping [16]. Furthermore, our extended analysis in naïve and memory human T cell subsets demonstrated that Compound 1 preferentially augments the activation and cytokine production of the memory CD8+ T cell compartment, suggesting the possibility that inhibition of HPK1 activity may disrupt the acquisition of CD8+T cell exhaustion. Moreover, these observations have immediate implications for cancer immunotherapy approaches, including combination strategies that rely on HPK1 inhibition as a T cell functional augmentation strategy in the context of immune checkpoint therapy resistance. Since it is now appreciated that CD8+ T cells play a disproportionately important role in the immunosurveillance and elimination of cancers [39], our results suggest that pharmacologic inhibition of HPK1 may preferentially augment memory CD8+ T cell responses and invigorate anti-tumor T cells. Therefore, inhibition of HPK1 as a monotherapy or in combination with anti-PD-1/PD-L1 immunotherapy may impair the acquisition of resistance to checkpoint blockade, especially in hypoxic and adenosine rich tumors [40, 41]. Although we observed that Compound 1 exhibited preferentially stronger effects on memory CD8+ T cells, the underlying mechanism for this enhanced activity remains to be fully elucidated.

Accumulation of metabolic immunosuppressive molecules such as adenosine and PGE_2_ in the TME is a hallmark of immunotherapy resistance because their binding to the adenosine A_2A_/A_2B_ and EP_4_ receptors increases intracellular cAMP, which impairs T-cell effector function [5, 40–42]. Intervening in these metabolic pathways could enhance anti-tumor immunity and immunotherapy [43, 44]. Many drugs targeting the adenosine and COX-2/PGE_2_/EP_4_ pathways have been developed to reverse cAMP-suppression and are under clinic assessment [45–48]. Our data demonstrated that a HPK1 inhibitor can revert both GPCR-cAMP and FSK-mediated suppression, suggesting broad utility of a HPK1 inhibitor to overcome many inhibitory stimuli, resulting in increased cAMP in T cells. GPCR-cAMP-signaling also mediates strong suppression in monocytes and DCs, and blockade of adenosine or PGE_2_ signaling could reverse suppression in these cell types [49–52]. It is currently unknown whether HPK1 inhibition can reverse cAMP-mediated suppression in monocytes and DCs.

HPK1 deficiency in DCs has been reported to enhance pro-inflammatory cytokine secretion and antigen presentation function [17]. However, Compound 1 did not increase co-stimulatory molecule expression and surprisingly decreased TNF-α secretion in the current studies. A developmental role of HPK1 in DC function was not observed by adding Compound 1 during mBMDC differentiation. However, we cannot exclude the possibility that the lack of effect of Compound 1 on DC might be specific to Compound 1. While Compound 1 is a potent HPK1 inhibitor in biochemical assays measuring inhibition of SLP-76 phosphorylation and in T-cell target engagement and functional assays, the biological pathways of HPK1 in DCs are less well understood, and biochemical and cell potency may be weaker or include scaffolding functions in addition to kinase activity. Given the variable effects of different HPK1 inhibitors in DCs, future HPK1 inhibitor optimization may require screening in both T cells and DCs to identify an optimal inhibitor for development.

In summary, we found that the pharmacological inhibition of HPK1 by competitive small molecule inhibitors enhanced T-cell activation and restored cAMP-mediated suppression in T cells, making these compounds reasonable candidates for development into an immunomodulatory drug. While Compound 1 is a potent and highly selective HPK1 inhibitor, it did not fully recapitulate the reported effects of HPK1 genetic deletion or kinase inactive knock-in [15–17]. Additional inhibitors with improved selectivity and enhanced potency profiles need to be identified, and *in vivo* assessments using tools with adequate pharmacokinetic properties and target engagement need to be performed in order to further validate the anti-tumor efficacy of HPK1 inhibitors.

## Supporting information

S1 Fig Compound 1 did not inhibit cell viability. CD4+ or CD8+ T cells were treated with Compound 1 or Vehicle and stimulated with anti-CD3/CD28 mAb for 24 hours (A) or 72 hours (B) and then were stained with fixable viability dyes (FVS510 or FVS780). Live CD4+ or CD8+ T cells among total hCD4 + or hCD8 + T cells were determined by flow cytometry. Data are from one experimental representative of at least three independent experiments.
(TIF)

S2 Fig Compound 1 effect on anti-CD3/CD28-stimulated cytokines in human CD4 + and CD8 + T cells at 72 hours. hCD4+ T cells (A) and hCD8+ T cells (B) were isolated from PBMC and stimulated with 0.25μg/ml anti-CD3 and anti-CD28 for 72h. IFN-γ, IL-2 and TNF-α secretion were measured from supernatant using the Mesoscale Discovery (MSD) ELISA-based assay platform. The data shown were representative from three independent experiments (3 different donors). *P<0.05, **P<0.01 ***P<0.001, ****P<0.0001, one-way ANOVA with post-test analysis compared to anti-CD3/anti-CD28 group.
(TIF)

S3 Fig Compound 1 augmented T lymphocyte activation. Flow cytometry dot plots of CD69, CD25, and CD71 staining in anti-CD3/CD28 mAb-stimulated CD4+ (A) and CD8+ (B) T lymphocytes treated with 0.0097 μM Compound 1 or untreated controls after 24 hours. Data were from 1 experimental representative (triplicate treatment) of at least 3 independent experiments.
(TIF)

S4 Fig Compound 1 effect on lymphocyte proliferation. hCD4+ T cells (A, left panel), naïve CD4+ T cells (A, middle panel), memory CD4+ T cells (A, right panel), hCD8+ T cells (B, left panel), naïve CD8+ T cells (B, middle panel) and memory CD8+ T cells (B, right panel) were labeled with CFSE and then stimulated with 0.25μg/ml anti-CD3 and anti-CD28 for 72h. % of divided cells were considered as proliferation rate (%). C. Splenocytes from OTI mice(C, left panel) or OTII (D, right panel) mice were treated with compound 1 at 0.1μM and stimulated with various concentration of OVA_257-264_(C, left panel) or OVA_323-339_(C, right panel) 72h. The frequency of Ki-67 positive CD4+ and CD8+ T lymphocytes was as shown in C. The data shown were representative from three independent experiments (3 different donors). *P<0.05, **P<0.01 ***P<0.001, ****P<0.0001, one-way ANOVA with post-test analysis compared to anti-CD3/anti-CD28 group or OVA_257-264_ or OVA_323-339_, respectively.
(TIF)

S5 Fig Flow cytometry dot plots of CD4+/CD8+ T cells among viable splenocytes before and after peptide stimulation. Splenocytes from OT-1 mice (A) were treated with 10,000 ng/mL OVA_257-264_ for 24 hours and 72 hours, and the percentage of CD4+ and CD8+ T cells among total live cells was calculated. Splenocytes from OT-II mice (B) were treated with 10,000 ng/mL OVA_323-339_ for 24 hours and 72 hours, and the percentage of CD4+ and CD8+ T cells among total live cells was calculated. Data were from 1 experimental representative (triplicate treatment) of at least 3 independent experiments.
(TIF)

S6 Fig Compound 1 restored cAMP-suppressed cytokines in anti-CD3/CD28-stimulated human CD4+ T cells. hCD4+ T cells were isolated from PBMC and treated with compound 1 W/O PGE2(A), or NECA (B) or FSK(C), and then stimulated with 0.5μg/ml anti-CD3 and anti-CD28 for 24 hours. IFN-γ, IL-2 and TNF-α secretion were measured from supernatant by the Mesoscale Discovery (MSD) ELISA-based assay platform. The data shown are representative from three independent experiments (3 different donors). *P<0.05, **P<0.01 ***P<0.001, ****P<0.0001, one-way ANOVA with post-test analysis compared to anti-CD3/anti-CD28 group.
(TIF)

S7 Fig Compound 1 effect on cytokine and activation during DC development. Bone marrow cells were differentiated into DC in the presence or absence of vehicle or various dose of Compound 1 for 6 days and stimulated with 0.2μg/ml LPS for another 24h. TNF-α and IL-6 production were measured using the Mesoscale Discovery (MSD) ELISA-based assay platform (A). Geometric mean fluorescent intensity (MFI) of cell surface activation markers was shown in B and D. The data shown are representative from three independent experiments. *P<0.05, **P<0.01 ***P<0.001, ****P<0.0001, one-way ANOVA with post-test analysis compared to LPS group.
(TIF)

## Abbreviations

MAP4K1: Mitogen-Activated Protein Kinase Kinase Kinase Kinase 1
HPK1: Hematopoietic Progenitor Kinase 1
TCR: T cell receptor
CFSE: Carboxyfluorescein succinimidyl ester
NSCLC: non-small cell lung cancer
GM-CSF: granulocyte macrophage colony stimulating factor
LPS: lipopolysaccharide
OT1: C57BL/6-Tg(TcrαTcrβ)1100Mjb/J
OT2: B6.Cg-Tg(TcrαTcrβ) 425Cbn/J
PGE_2_: Prostaglandin E_2_
NECA: 5’-N-Ethylcarboxamido adenosine
PBMC: peripheral blood mononuclear cell
FSK: forskolin

## References

[1] Kim R, Emi M, Tanabe K. Cancer immunoediting from immune surveillance to immune escape. Immunol. 2007;121(1):1–14. 10.1111/j.1365-2567.2007.02587.x . 17386080 PMC2265921

[2] Hkslil DN, Smith EL, Brentiens RJ, Wolchok JD. The future of cancer treatment: immunomodulation, CARs and combination immunotherapy. Nature Rev Clin Oncol. 2016;13(5):273–290. 10.1038/nrclinonc.2016.25 . 26977780 PMC5551685

[3] Chen DS, Mellman I. Oncology meets immunology: the cancer-immunity cycle. Immunity. 2013; 39:1–10. 10.1016/j.immuni.2013.07.012 . 23890059

[4] Lee HT, Lee SH, Heo YS. Molecular interactions of antibody drugs targeting PD-1, PD-L1, and CTLA-4 in immuno-oncology. Molecules. 2019;24(6):1190–1205. 10.3390/molecules24061190 . 30917623 PMC6470598

[5] Sharma P, Hu-Lieskovan S, Wargo JA, Ribas A. Primary, adaptive, and acquired resistance to cancer immunotherapy. Cell. 2017;168(4):707–723. 10.1016/j.cell.2017.01.017 . 28187290 PMC5391692

[6] Sawasdikosol S, Zha R, Yang B, Burakoff S. HPK1 as a novel target for cancer immunotherapy. Immunol Res. 2012; 54:262–265. 10.1007/s12026-012-8319-1 . 22477524

[7] Hu MC, Qiu WR, Wang X, Meyer CF, Tan TH. Human HPK1, a novel human hematopoietic progenitor kinase that activates the JNK/SAPK kinase cascade. Genes Devel. 1996; 10:2251–2264. 10.1101/gad.10.18.2251 . 8824585

[8] Kiefer F, Tibbles LA, Anafi M, Janssen A, Zanke BW et al, HPK1, a hematopoietic protein kinase activating the SAPK/JNK pathway. EMBO J. 1996; 15:7013–7025. . 9003777 PMC452527

[9] Brenner D, Brechmann M, Röhling S, Tapernoux M, Mock T et al, Phosphorylation of CARMA1 by HPK1 is critical for NF-kappaB activation in T cells. Proc Nat Acad Sci USA. 2009;106(34):14508–14513. 10.1073/pnas.0900457106 . 19706536 PMC2732850

[10] Wang X, Li JP, Chiu LL, Lan JL, Chen DY et al, Attenuation of T cell receptor signaling by serine phosphorylation-mediated lysine 30 ubiquitination of SLP-76 protein. J Biol Chem. 2012; 287(41):34091–34100. 10.1074/jbc.M112.371062 . 22902619 PMC3464518

[11] Wang X, Li JP, Kuo HK, Chiu LL, Dement GA et al, Down-regulation of B cell receptor signaling by hematopoietic progenitor kinase 1 (HPK1)-mediated phosphorylation and ubiquitination of activated B cell linker protein (BLNK). J Biol Chem. 2012;287(14):11037–11048. 10.1074/jbc.M111.310946 . 22334673 PMC3322877

[12] Di Bartolo V, Montagne B, Salek M, Jungwirth B, Carrette F et al, A novel pathway down-modulating T cell activation involves HPK-1-dependent recruitment of 14-3-3 proteins on SLP-76. J Exp Med. 2007;204(3):681–91. 10.1084/jem.20062066 . 17353368 PMC2137917

[13] Ling P, Meyer CF, Redmond LP, Shui JW, Davis B, Rich RR, et al Involvement of hematopoietic progenitor kinase 1 in T cell receptor signaling. J Biol Chem. 2001;276(22):18908–18914. 10.1074/jbc.M101485200 . 11279207

[14] Shui JW, Boomer JS, Han J, Xu J, Dement GA, Zhou G, et al Hematopoietic progenitor kinase 1 negatively regulates T cell receptor signaling and T cell-mediated immune responses. Nature Immunol. 2007;8(1):84–91. 10.1038/ni1416 . 17115060

[15] Liu J, Curtin J, You D, Hillerman S, Li-Wang B, Eraslan R, et al Critical role of kinase activity of hematopoietic progenitor kinase 1 in anti-tumor immune surveillance. PLOS ONE. 2019;14(3): e0212670 10.1371/journal.pone.0212670 . 30913212 PMC6435129

[16] Hernandez S, Qing J, Thibodeau RH, Du X, Park S, Lee HM, et al The kinase activity of hematopoietic progenitor kinase 1 is essential for the regulation of T cell function. Cell Rep. 2018;25(1):80–94. 10.1016/j.celrep.2018.09.012 30282040

[17] Alzabin S, Bhardwaj N, Kiefer F, Sawasdikosol S, Burakoff S. Hepatopoietic progenitor kinase 1 is a negative regulator of dendritic cell activation. J Immunol. 2009;182(10):6187–94. 10.4049/jimmunol.0802631 . 19414772

[18] Kalinski P. Regulation of immune responses by prostaglandin E_2_. J Immunol. 2012;188(1):21–28. 10.4049/jimmunol.1101029 . 22187483 PMC3249979

[19] Arab S, Hadiati J. Adenosine blockade in tumor microenvironment and improvement of cancer immunotherapy. Immune Network. 2019;19(4): e23 10.4110/in.2019.19.e23 . 31501711 PMC6722273

[20] Sawasdikosol S, Pyarajan S, Alzabin S, Matejovic G, Burakoff SJ. Prostaglandin E2 activates HPK1 kinase activity via a PKA-dependent pathway. J Biol Chem. 2007;282(48):34693–34699. 10.1074/jbc.M707425200 . 17895239

[21] Alzabin S, Pyarajan S, Yee H, Kiefer F, Suzuki A, Burakoff S, et al Hematopoietic progenitor kinase 1 is a critical component of prostaglandin E2-mediated suppression of the anti-tumor immune response. Cancer Immunol Immunother. 2010;59(3):419–429. 10.1007/s00262-009-0761-0 . 19787351 PMC2798028

[22] Stuart T, Butler A, Hoffman P, Hafemeister C, Papalexi E, et al, Comprehensive Integration of Single-Cell Data. Cell. 2019; 177: 1888–1902. 10.1016/j.cell.2019.05.031 . 31178118 PMC6687398

[23] Georgiev P, Wang Y, Muise ES, Bandi ML, Blumenschein W, et al, BET Bromodomain Inhibition Suppresses Human T Cell Function. Immunohorizons. 2019; 3(7):294–305. 10.4049/immunohorizons.1900037 . 31356159

[24] Zhang Q, Ding S, Zhang H. Interactions between hematopoietic progenitor kinase 1 and its adaptor proteins (Review). Molec Med Rep. 2017;16(5):6472–6482. 10.3892/mmr.2017.7494 . 28901492

[25] Chan B, Drobnick J, Gazzard L, Heffron T, Liang J, Malhotra S et al Isoquinolines as inhibitors of HPK1. PCT Patent Publication. WO 2018183964 A1 2018.

[26] Lacey BM, Xu Z, Chai X, Laskey J, Fradera X et al, Development of High-Throughput Assays for Evaluation of Hematopoietic Progenitor Kinase 1 Inhibitors. SLAS Discov. 2020; 2472555220952071. 10.1177/2472555220952071 . 32844715

[27] Ni L, Lu J. Interferon gamma in cancer immunotherapy. Cancer Med. 2018;7(9):4509–4516. 10.1002/cam4.1700 . 30039553 PMC6143921

[28] Rodriguez G, Ross JA, Nagy ZS, Kirken RA. Forskolin-inducible cAMP pathway negatively regulates T-cell proliferation by uncoupling the interleukin-2 receptor complex. J Biol Chem. 2013;288(10):7137–7146. 10.1074/jbc.M112.408765 . 23341462 PMC3591623

[29] Cristescu R, Mogg R, Ayers M, Albright A, Murphy E et al, Pan-tumor genomic biomarkers for PD-1 checkpoint blockade-based immunotherapy. Science. 2018;362(6411): eaar3593 aar3593. Erratum in: Science. 2019;363(6430): 10.1126/science.aar3593 . 30309915 PMC6718162

[30] Ciciola P, Cascetta P, Bianco C, Formisano L, Bianco R. Combining Immune Checkpoint Inhibitors with Anti-Angiogenic Agents. J Clin Med. 2020;9(3):675 10.3390/jcm9030675 . 32138216 PMC7141336

[31] Rotte A. Combination of CTLA-4 and PD-1 blockers for treatment of cancer. J Exp Clin Cancer Res. 2019;38(1):255 10.1186/s13046-019-1259-z . 31196207 PMC6567914

[32] Seidel JA, Otsuka A, Kabashima K. Anti-PD-1 and Anti-CTLA-4 Therapies in Cancer: Mechanisms of Action, Efficacy, and Limitations. Front Oncol. 2018; 8:86 10.3389/fonc.2018.00086 . 29644214 PMC5883082

[33] Diab A, Tannir NM, Bentebibel SE, Hwu P, Papadimitrakopoulou V et al, Bempegaldesleukin (NKTR-214) plus Nivolumab in Patients with Advanced Solid Tumors: Phase I Dose-Escalation Study of Safety, Efficacy, and Immune Activation (PIVOT-02). *Cancer Discov*. 2020;10(8):1158–1173. 10.1158/2159-8290.CD-19-1510 . 32439653

[34] Khushalani NI, Diab A, Ascierto PA, Larkin J, Sandhu S et al, Bempegaldesleukin plus nivolumab in untreated, unresectable or metastatic melanoma: Phase III PIVOT IO 001 study design [published online ahead of print, 2020 Jul 29]. Future Oncol. 2020;10.2217/fon-2020-0351. 10.2217/fon-2020-0351 . 32723187

[35] Kumar S, Principe DR, Singh SK, Viswakarma N, Sondarva G, Rana B, et al Mitogen-activated protein kinase inhibitors and T-cell–dependent immunotherapy in cancer. Pharmaceuticals (Basel). 2020;13(1):9 10.3390/ph13010009 . 31936067 PMC7168889

[36] Arnold R, Patzak IM, Neuhaus B, Vancauwenbergh S, Veillette A, Van Lint J, et al Activation of hematopoietic progenitor kinase 1 involves relocation, autophosphorylation, and transphosphorylation by protein kinase D1. Mol Cell Biol. 2005;25(6):2364–2383. 10.1128/MCB.25.6.2364-2383.2005 15743830 PMC1061595

[37] Sawasdikosol S, Burakoff S. The structure of HPK1 kinase domain: to boldly go where no immuno-oncology drugs have gone before. Structure. 2019;27(1):1‐3. 10.1016/j.str.2018.12.009 30605659

[38] Hoelder S, Clarke PA, Workman P. Discovery of small molecule cancer drugs: successes, challenges and opportunities. Mol Oncol. 2012;6(2):155–176. 10.1016/j.molonc.2012.02.004 . 22440008 PMC3476506

[39] Pluhar GE, Pennell CA, Olin MR. CD8⁺ T Cell-Independent Immune-Mediated Mechanisms of Anti-Tumor Activity. Crit Rev Immunol. 2015;35(2):153–72. 10.1615/critrevimmunol.2015013607 . 26351148 PMC4870933

[40] Sitkovsky MV. Lessons from the A2A Adenosine Receptor Antagonist-Enabled Tumor Regression and Survival in Patients with Treatment-Refractory Renal Cell Cancer. Cancer Discov. 2020;10(1):16–19. 10.1158/2159-8290.CD-19-1280 . 31919119

[41] Sitkovsky MV. Sufficient numbers of anti-tumor T cells is a condition of maximal efficacy of anti-hypoxia-A2-adenosinergic drugs during cancer immunotherapy. Curr Opin Pharmacol. 2020; 53:98–100. 10.1016/j.coph.2020.07.011 . 32861959

[42] Greenhough A, Smartt H, Moore AE, Roberts HR, Williams AC, Paraskeva C, et al The COX-2/PGE_2_ pathway: key roles in the hallmarks of cancer and adaptation to the tumour microenvironment. Carcinogenesis. 2009;30(3):377–386. 10.1093/carcin/bgp014 . 19136477

[43] Li X, Wenes M, Romero P, Huang SC, Fendt SM, Ho PC. Navigating metabolic pathways to enhance antitumour immunity and immunotherapy. Nature Rev Clin Oncol. 2019;16(7):425–441. 10.1038/s41571-019-0203-7 . 30914826

[44] Zelenay S, van der Veen AG, Böttcher JP, Snelgrove KJ, Rogers N et al, Cyclooxygenase-Dependent Tumor Growth through Evasion of Immunity. Cell. 2015;162(6):1257–70. 10.1016/j.cell.2015.08.015 . 26343581 PMC4597191

[45] Beatty GL, Gladney WL. Immune escape mechanisms as a guide for cancer immunotherapy. Clin Cancer Res. 2015;21(4):687–692. 10.1158/1078-0432.CCR-14-1860 . 25501578 PMC4334715

[46] Vigano S, Alatzoglou D, Irving M, Ménétrier-Caux C, Caux C, Romero P, et al Targeting adenosine in cancer immunotherapy to enhance T-cell function. Frontiers Immunol. 2019; 10:925 10.3389/fimmu.2019.00925 . 31244820 PMC6562565

[47] Leone RD, Emens LA. Targeting adenosine for cancer immunotherapy. J Immunother Cancer. 2018;6(1):57 10.1186/s40425-018-0360-8 29914571 PMC6006764

[48] Majumder M, Nandi P, Omar A, Ugwuagbo KC, Lala PK. EP4 as a therapeutic target for aggressive human breast cancer. Int J Mol Sci. 2018;19(4):1019 10.3390/ijms19041019 . 29596308 PMC5979567

[49] Sciaraffia E, Riccomi A, Lindstedt R, Gesa V, Cirelli E, Patrizio M, et al Human monocytes respond to extracellular cAMP through A2A and A2B adenosine receptors. J Leukocyte Biol. 2014;96(1):113–122. 10.1189/jlb.3A0513-302RR . 24652540 PMC4056277

[50] Novitskiy SV, Ryzhov S, Zaynagetdinov R, Goldstein AE, Huang Y, Tikhomirov OY, et al Adenosine receptors in regulation of dendritic cell differentiation and function. Blood. 2018;112(5):1822–1831. 10.1182/blood-2008-02-136325 . 18559975 PMC2518889

[51] Harizi H, Grosset C, Gualde N. Prostaglandin E2 modulates dendritic cell function via EP2 and EP4 receptor subtypes. J Leukocyte Biol. 2003;73(6):756–763. 10.1189/jlb.1002483 . 12773508

[52] Kashmiry A, Tate R, Rotondo G, Davidson J, Rotondo D. The prostaglandin EP4 receptor is a master regulator of the expression of PGE2 receptors following inflammatory activation in human monocytic cells. Biochim Biophys Acta Mol Cell Biol Lipid. 2018;1863(10):1297–1304. 10.1016/j.bbalip.2018.07.003 . 30053598

