# Supplementary figures and images for: Pharmacological inhibition of hematopoietic progenitor kinase 1 positively regulates T-cell function
Source: PLoS One. 2020 Dec 3;15(12):e0243145. doi: 10.1371/journal.pone.0243145 (PMC7714195; doi:10.1371/journal.pone.0243145)

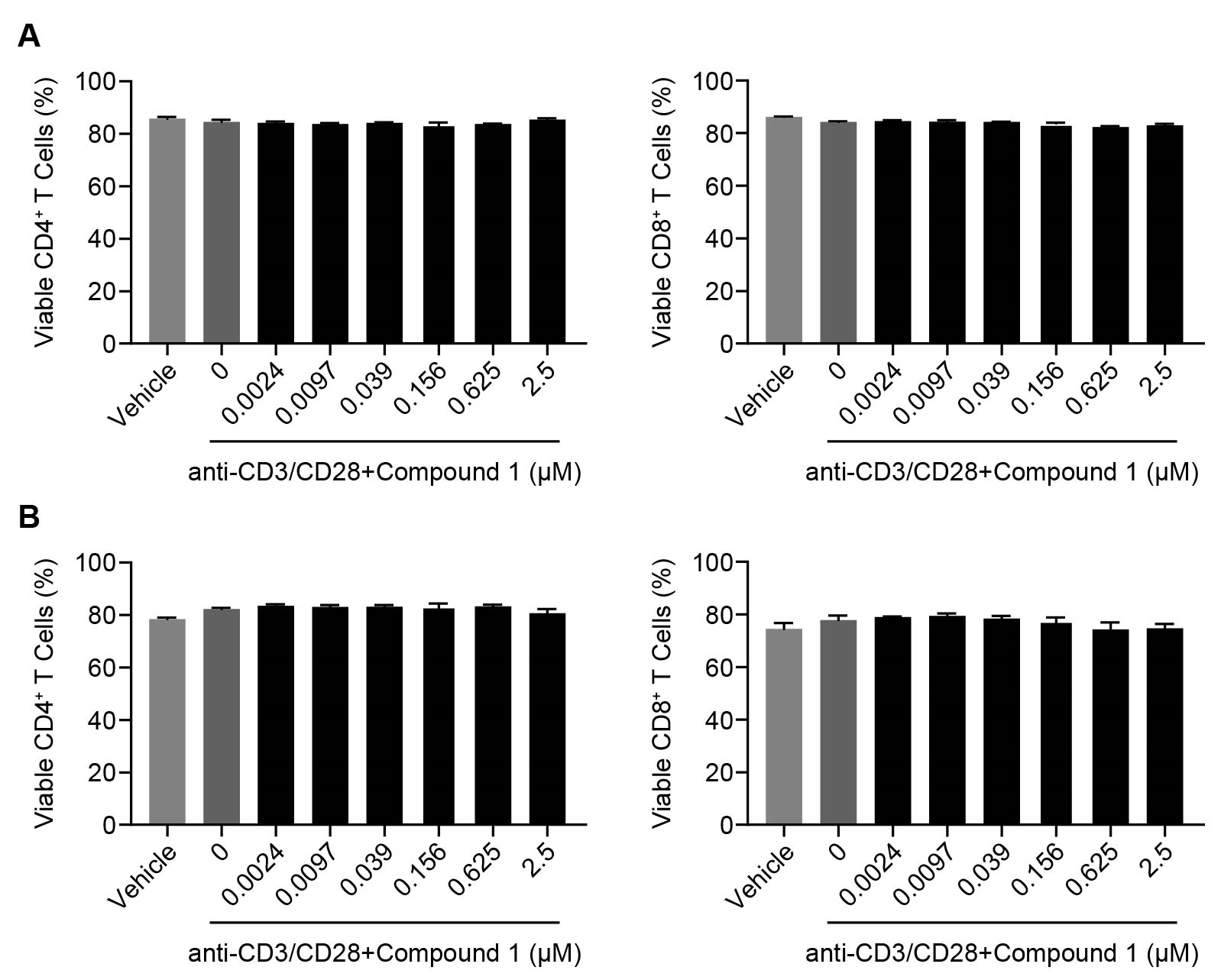

Supplement: S1 Fig — CD4+ or CD8+ T cells were treated with Compound 1 or Vehicle and stimulated with anti-CD3/CD28 mAb for 24 hours (A) or 72 hours (B) and then were stained with fixable viability dyes (FVS510 or FVS780). Live CD4+ or CD8+ T cells among total hCD4 + or hCD8 + T cells were determined by flow cytometry. Data are from one experimental representative of at least three independent experiments. (TIF) [file pone.0243145.s001.tif]

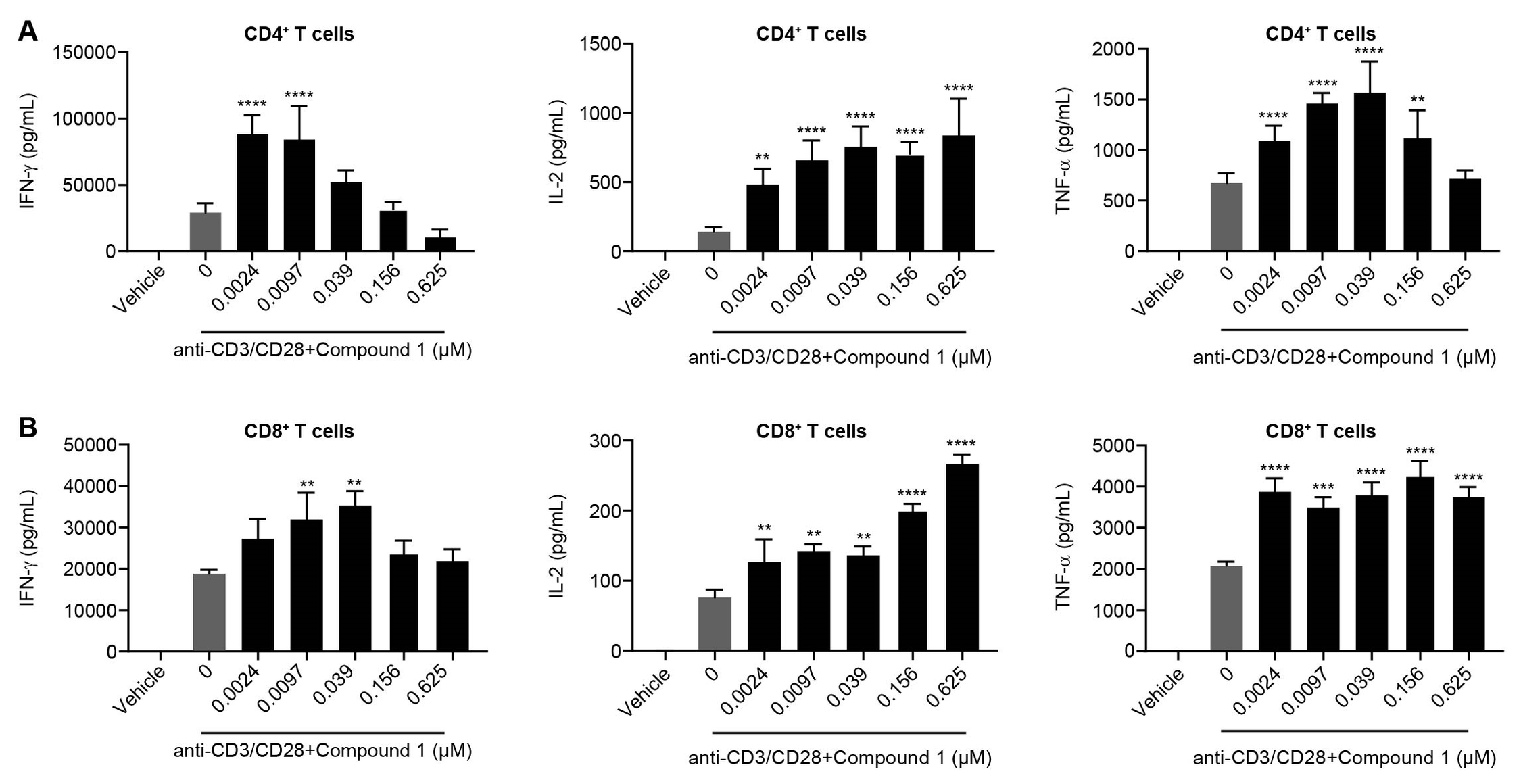

Supplement: S2 Fig — hCD4+ T cells (A) and hCD8+ T cells (B) were isolated from PBMC and stimulated with 0.25μg/ml anti-CD3 and anti-CD28 for 72h. IFN-γ, IL-2 and TNF-α secretion were measured from supernatant using the Mesoscale Discovery (MSD) ELISA-based assay platform. The data shown were representative from three independent experiments (3 different donors). *P<0.05, **P<0.01 ***P<0.001, ****P<0.0001, one-way ANOVA with post-test analysis compared to anti-CD3/anti-CD28 group. (TIF) [file pone.0243145.s002.tif]

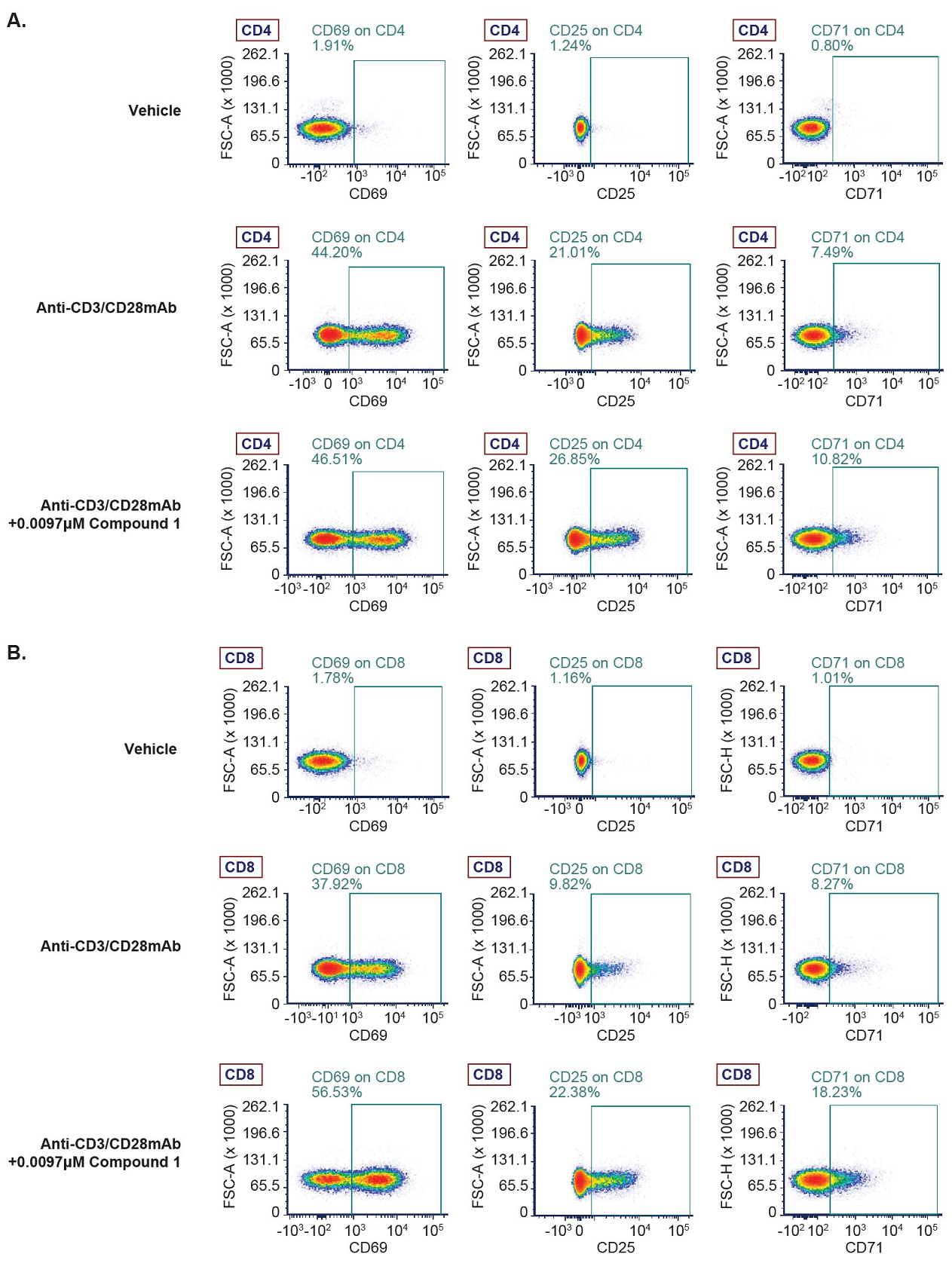

Supplement: S3 Fig — Flow cytometry dot plots of CD69, CD25, and CD71 staining in anti-CD3/CD28 mAb-stimulated CD4+ (A) and CD8+ (B) T lymphocytes treated with 0.0097 μM Compound 1 or untreated controls after 24 hours. Data were from 1 experimental representative (triplicate treatment) of at least 3 independent experiments. (TIF) [file pone.0243145.s003.tif]

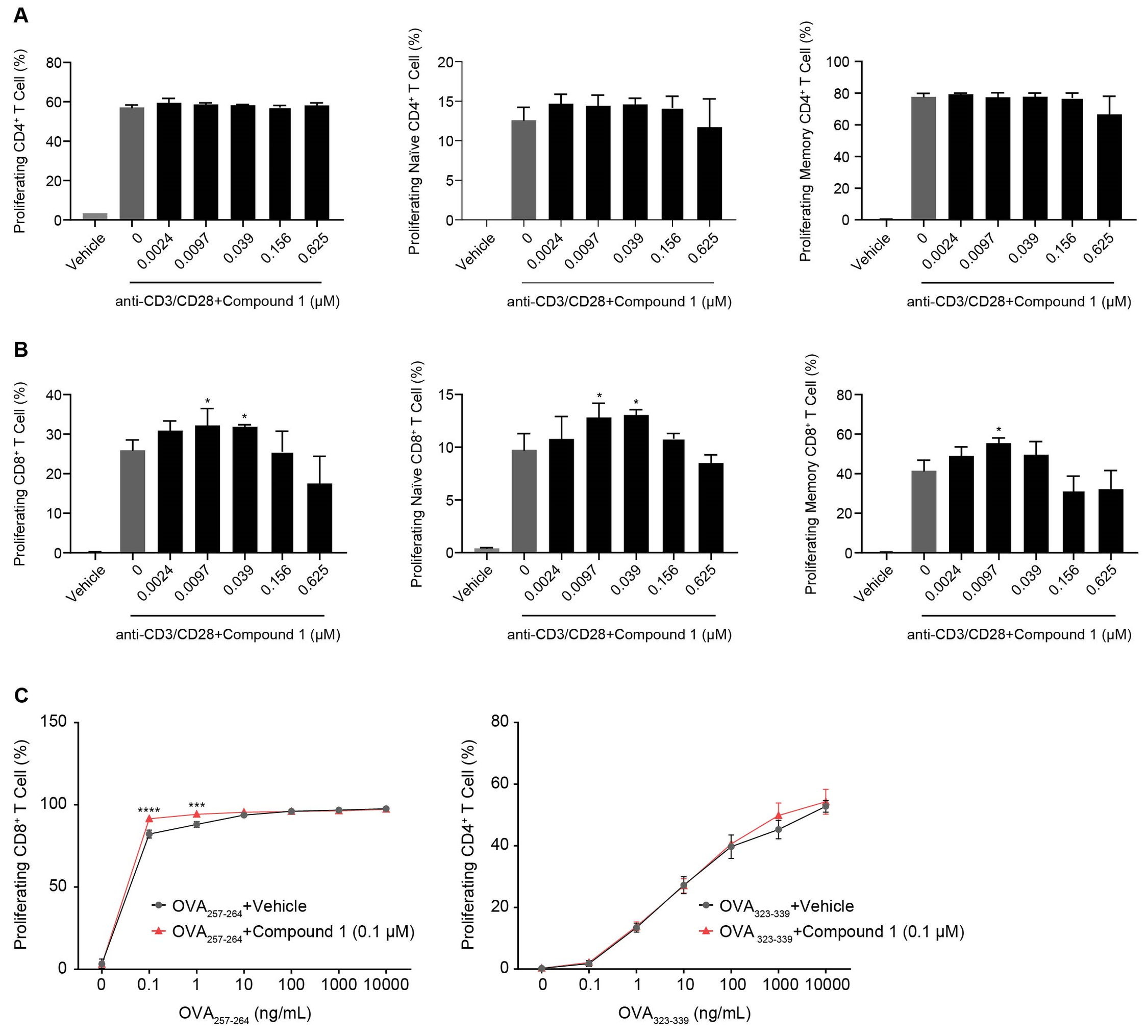

Supplement: S4 Fig — hCD4+ T cells (A, left panel), naïve CD4+ T cells (A, middle panel), memory CD4+ T cells (A, right panel), hCD8+ T cells (B, left panel), naïve CD8+ T cells (B, middle panel) and memory CD8+ T cells (B, right panel) were labeled with CFSE and then stimulated with 0.25μg/ml anti-CD3 and anti-CD28 for 72h. % of divided cells were considered as proliferation rate (%). C. Splenocytes from OTI mice(C, left panel) or OTII (D, right panel) mice were treated with compound 1 at 0.1μM and stimulated with various concentration of OVA257-264(C, left panel) or OVA323-339(C, right panel) 72h. The frequency of Ki-67 positive CD4+ and CD8+ T lymphocytes was as shown in C. The data shown were representative from three independent experiments (3 different donors). *P<0.05, **P<0.01 ***P<0.001, ****P<0.0001, one-way ANOVA with post-test analysis compared to anti-CD3/anti-CD28 group or OVA257-264 or OVA323-339, respectively. (TIF) [file pone.0243145.s004.tif]

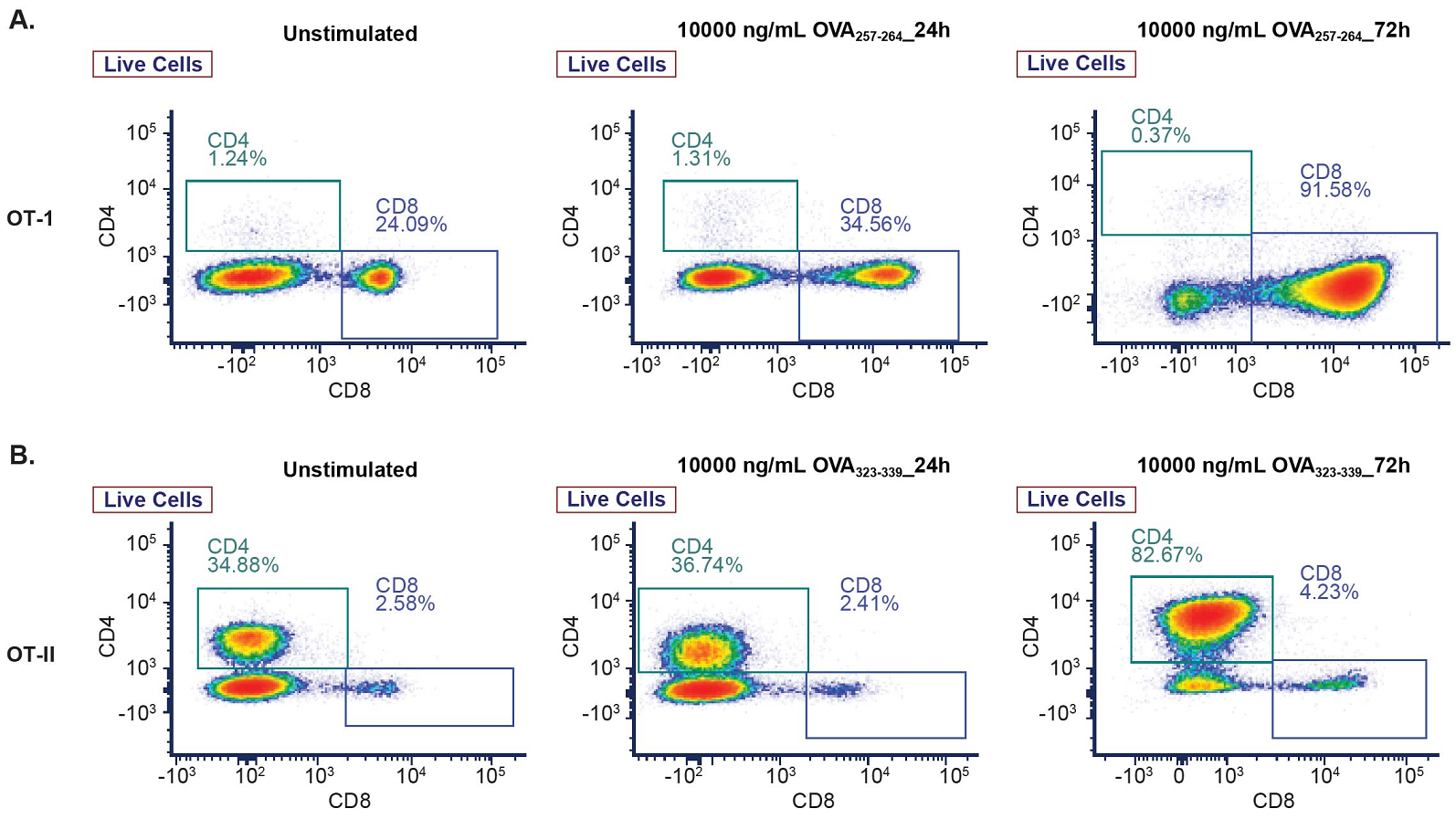

Supplement: S5 Fig — Splenocytes from OT-1 mice (A) were treated with 10,000 ng/mL OVA257-264 for 24 hours and 72 hours, and the percentage of CD4+ and CD8+ T cells among total live cells was calculated. Splenocytes from OT-II mice (B) were treated with 10,000 ng/mL OVA323-339 for 24 hours and 72 hours, and the percentage of CD4+ and CD8+ T cells among total live cells was calculated. Data were from 1 experimental representative (triplicate treatment) of at least 3 independent experiments. (TIF) [file pone.0243145.s005.tif]

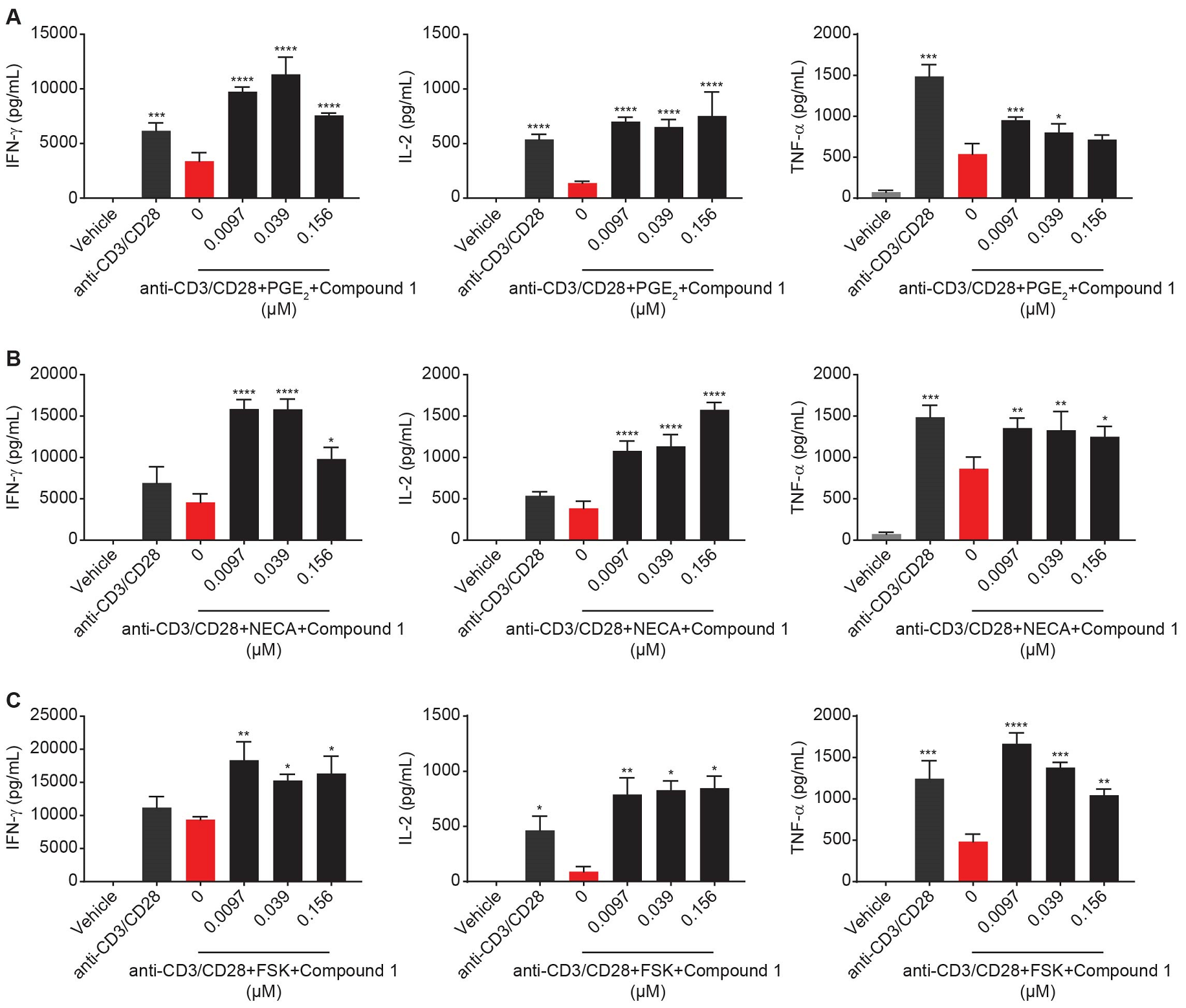

Supplement: S6 Fig — hCD4+ T cells were isolated from PBMC and treated with compound 1 W/O PGE2(A), or NECA (B) or FSK(C), and then stimulated with 0.5μg/ml anti-CD3 and anti-CD28 for 24 hours. IFN-γ, IL-2 and TNF-α secretion were measured from supernatant by the Mesoscale Discovery (MSD) ELISA-based assay platform. The data shown are representative from three independent experiments (3 different donors). *P<0.05, **P<0.01 ***P<0.001, ****P<0.0001, one-way ANOVA with post-test analysis compared to anti-CD3/anti-CD28 group. (TIF) [file pone.0243145.s006.tif]

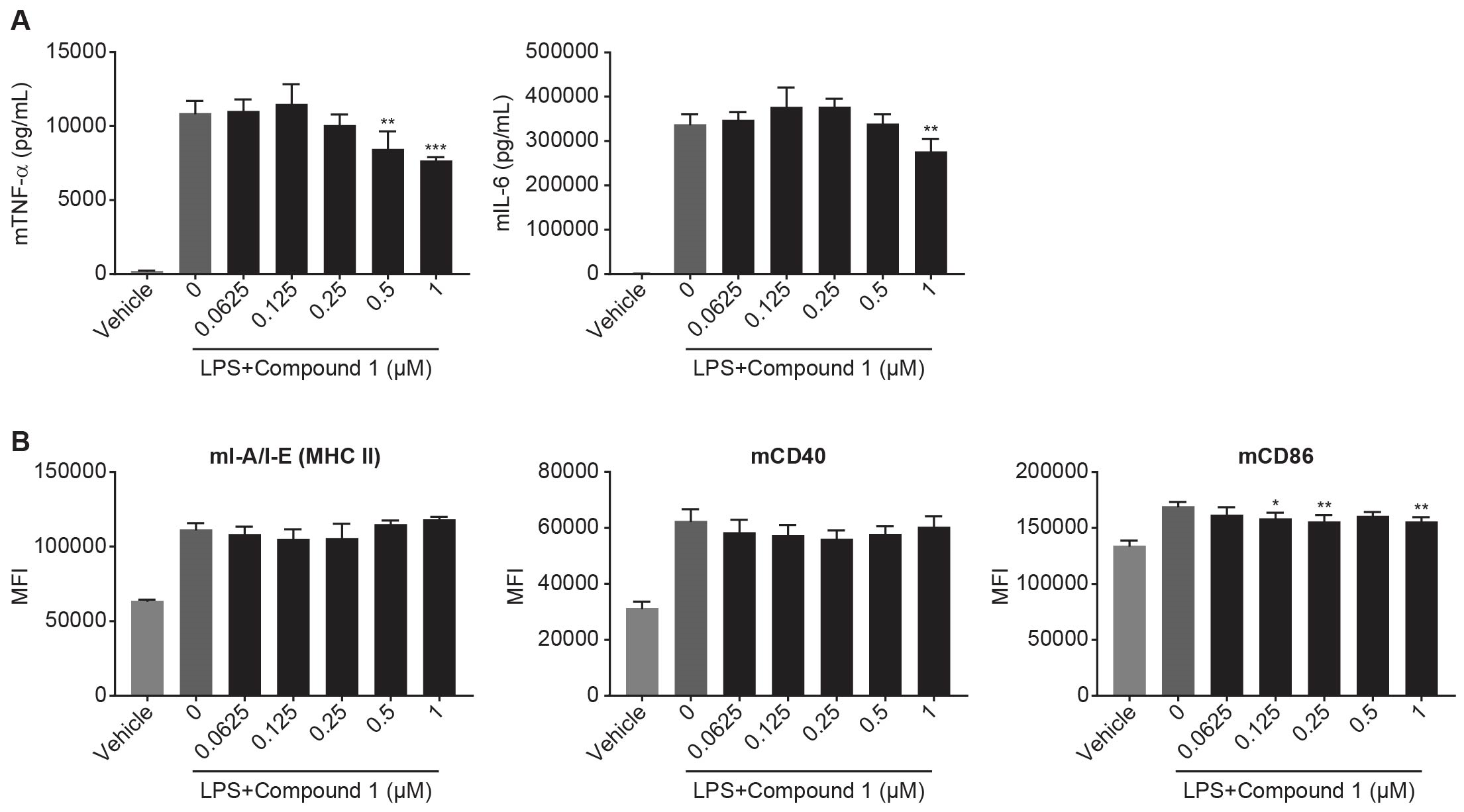

Supplement: S7 Fig — Bone marrow cells were differentiated into DC in the presence or absence of vehicle or various dose of Compound 1 for 6 days and stimulated with 0.2μg/ml LPS for another 24h. TNF-α and IL-6 production were measured using the Mesoscale Discovery (MSD) ELISA-based assay platform (A). Geometric mean fluorescent intensity (MFI) of cell surface activation markers was shown in B and D. The data shown are representative from three independent experiments. *P<0.05, **P<0.01 ***P<0.001, ****P<0.0001, one-way ANOVA with post-test analysis compared to LPS group. (TIF) [file pone.0243145.s007.tif]
